# Intervertebral disc-intrinsic Hedgehog signaling maintains disc cell phenotypes and prevents disc degeneration through both cell autonomous and non-autonomous mechanisms

**DOI:** 10.1007/s00018-023-05106-x

**Published:** 2024-02-03

**Authors:** Lei Zhang, Siyuan Hu, Chunmei Xiu, Meng Li, Yixin Zheng, Rui Zhang, Bin Li, Jianquan Chen

**Affiliations:** 1 Department of Clinical Medicine, Key Laboratory of Novel Targets and Drug Study for Neural Repair of Zhejiang Province, School of Medicine, Hangzhou City University, Hangzhou, 310015 Zhejiang China; 2 Orthopedic Institute, Suzhou Medical College, Soochow University, Suzhou, 215006 Jiangsu China

**Keywords:** Low back pain, Shh signaling, Intervertebral disc homeostasis, Gli3 repressor, Dis cell differentiation, Chondrocyte-like cells

## Abstract

Intervertebral disc degeneration is closely related to abnormal phenotypic changes in disc cells. However, the mechanism by which disc cell phenotypes are maintained remains poorly understood. Here, Hedgehog-responsive cells were found to be specifically localized in the inner annulus fibrosus and cartilaginous endplate of postnatal discs, likely activated by Indian Hedgehog. Global inhibition of Hedgehog signaling using a pharmacological inhibitor or *Agc1-CreER*^*T2*^*-*mediated deletion of *Smo* in disc cells of juvenile mice led to spontaneous degenerative changes in annulus fibrosus and cartilaginous endplate accompanied by aberrant disc cell differentiation in adult mice. In contrast, *Krt19-CreER*-mediated deletion of *Smo* specifically in nucleus pulposus cells led to healthy discs and normal disc cell phenotypes. Similarly, age-related degeneration of nucleus pulposus was accelerated by genetic inactivation of Hedgehog signaling in all disc cells, but not in nucleus pulposus cells. Furthermore, inactivation of *Gli2* in disc cells resulted in partial loss of the vertebral growth plate but otherwise healthy discs, whereas deletion of *Gli3* in disc cells largely corrected disc defects caused by *Smo* ablation in mice. Taken together, our findings not only revealed for the first time a direct role of Hedgehog-Gli3 signaling in maintaining homeostasis and cell phenotypes of annuls fibrosus and cartilaginous endplate, but also identified disc-intrinsic Hedgehog signaling as a novel non-cell-autonomous mechanism to regulate nucleus pulposus cell phenotype and protect mice from age-dependent nucleus pulposus degeneration. Thus, targeting Hedgehog signaling may represent a potential therapeutic strategy for the prevention and treatment of intervertebral disc degeneration.

**Supplementary Information:**

The online version contains supplementary material available at 10.1007/s00018-023-05106-x.

## Introduction

Low back pain (LBP), a prevalent musculoskeletal disease, represents the leading cause of disability worldwide [1]. Among its various etiological factors, intervertebral disc (IVD) degeneration (IDD) is considered as the major one. Unfortunately, there are no effective treatment that can reverse or delay IDD progression [2]. The IVD comprises a central nucleus pulposus (NP), a surrounding annulus fibrosus (AF), and a cartilaginous endplate (CEP) located below and above NP/AF [3, 4]. The NP and AF function to resist compression and tensile forces, respectively, whereas the CEP integrates IVD into the two neighboring vertebrae and delivers nutrients to the entire IVD. Under pathological conditions such as aging, NP cells undergo transition into chondrocyte-like cells or apoptosis, resulting in excessive production of chondrogenic and/or fibrotic matrix at the expense of hydrated gelatinous NP matrix [4–8]. These phenotypic changes in NP tissues in turn lead to degeneration of the entire IVD. Besides NP cells, IDD can also originate from abnormality in AF cells [4, 9]. However, the mechanism by which NP or AF cell phenotypes are maintained during IVD growth and homeostasis, and its relevance to the pathogenesis of IDD remain poorly understood.

Hedgehog (Hh) signaling is an evolutionarily conserved developmental pathway that plays diverse roles in tissue development and homeostasis [10]. In mammals, the Hh signaling pathway is initiated by one of three Hh ligands, transduced by Smoothened (Smo), and mediated intracellularly by the Gli family of transcriptional effectors, including Gli1, Gli2, and Gli3 [10, 11]. Gli2 and Gli3 are main effectors of Hh signaling, whereas Gli1, a direct target of Hh signaling, usually acts as an amplifier of the transcriptional response of Hh signaling [10–12]. Although Gli2 and Gli3 can exist as full-length activators (GliA) or truncated repressors (GliR), Gli2 appears to function predominantly as a transcriptional activator (Gli2A), whereas Gli3 primarily acts as a repressor (Gli3R) [11, 12]. Hh signaling promotes transcription of target genes by activating GliA and/or inhibiting GliR [10–12].

Hh signaling has been implicated in IVD growth and homeostasis in postnatal mice [13]. NP cells were found to express high level of *Shh* during the neonate period [14, 15]. Genetic deletion of *Shh* in mice at postnatal day (P)5, not only impaired proliferation of NP cells, but also affected differentiation of NP, AF and CEP cells, resulting in abnormal structures of both NP and AF tissues by P10 [14]. Consistently, pharmacological inhibition of Hh signaling in cultured P4 discs with cyclopamine, a small molecule inhibitor of Smo, led to the similar phenotypes to those caused by *Shh* deletion [14]. On the other hand, *Ihh* was detected in prehypertrophic chondrocytes of postnatal growth plates [16], whereas conditional ablation of *Ihh* from newborn mice using *Col2a1-CreER* caused an enlargement of NP tissue and a partial loss of AF by P14 [16]. Furthermore, systemic inhibition of Hh signaling with GDC-0449 (also named as vismodegib, and referred to as GDC hereafter), a potent inhibitor of Smo, in 8-week-old mice progressively led to some degenerative changes in IVDs [17]. The above studies have clearly demonstrated that Hh signaling plays important and complex roles in regulating IVD growth and homeostasis in postnatal mice. However, studies involving pharmacological inhibition of Smo and genetic ablation of *Shh* or *Ihh* did not address whether the effects of Hh signaling on postnatal IVDs is mediated via its activity in disc cells or via a non-cell autonomous mechanism. Moreover, it is still unknown which Gli protein mediates the function of Hh signaling in the context of IVD growth and homeostasis. In addition, the activity of Hh signaling in postnatal IVDs was shown to decrease with age [18, 19]. However, it remains unclear whether the reduced Hh signaling activity in disc cells contributes to age-related degenerative changes in IVDs.

In this study, we used both pharmacological and genetic tools to dissect the in vivo role of Hh signaling in IVD growth and maintenance in juvenile mice. Our results demonstrated that Hh signaling maintains disc cell phenotypes and prevents disc degeneration through both cell autonomous and non-autonomous mechanisms.

## Materials and methods

### Animals and treatments

*Agc1-CreER*^*T2*^, *Krt19-CreER*, *Gli1-LacZ*, *R26-tdTomato*, and mice carrying conditional alleles *of Smo* (*Smo*^*c*^ mice), *Gli2* (*Gli2*^*flox*^ mice), and *Gli3* (*Gli3*^*flox*^ mice) were described previously [20–26]. For conditional deletion of *Smo* in intervertebral disc cells or nucleus pulposus cells, *Agc1-CreER*^*T2*^; *Smo*^*c/c*^ or *Krt19-CreER; Smo*^*c/c*^ mice were obtained by crossing *Smo*^*c/c*^ mice with *Agc1-CreER*^*T2*^ or *Krt19-CreER* mice, respectively. For IVD-specific deletion of *Gli2*, *Agc1-CreER*^*T2*^; *Gli2*^*flox/flox*^ mice were generated by crossing *Gli2*^*flox/flox*^ mice with *Agc1-CreER*^*T2*^ mice. To evaluate the targeting efficiency and specificity of *Krt19-CreER* in the IVDs, *Krt19-CreER* mice were bred with *R26-tdTomato* reporter mice to produce *Krt19-CreER*; *R26-tdTomato* mice. All above mice and their littermate controls were injected with tamoxifen at 2 weeks of age according to our previously described procedures [27]. To evaluate the effect of pharmacological inhibition of Hh signaling on the health of IVDs, ten 2-week-old C57BL/6 mice were randomly divided into two groups, and orally administered with either 50 mg/kg vismodegib (GDC) or vehicle solution (vehicle) as described previously [28]. All mice were raised in a specific pathogen free (SPF) animal barrier facility.

### Micro-computer tomography (µCT) analysis

µCT analyses were performed as previously described [29]. The height of the intervertebral discs or vertebrae was then determined by averaging the measurements at the left, midline, and right regions of the corresponding discs or vertebrae. Disc height index (DHI) was calculated following the approach described previously [30, 31]. The lower lumbar levels (L4-6) from each group were used for analysis of the IVD height, vertebral height, DHI, and vertebral trabecular bone parameters.

### Histological analyses

Paraffin/frozen section preparation, Hematoxylin/eosin (H&E) staining, and Safranin O/Fast green staining were performed following the procedures described previously [27]. AF width, NP cell area (Ar. NP cells) and the percentage of NP cell area out of total NP space area (Ar. NP cells/Ar. NP space) were determined from mid-coronal sections of lower lumbar IVDs using the ImageJ software. To assess the degeneration of IVD in the AF and NP compartments, the mid-coronal sections of lower lumbar IVDs were graded for the AF and NP histopathological scores according to the scoring system described by Tam et al. [32]. Degeneration of IVD in the CEP compartment was analyzed using the CEP scoring system developed by Boos et al. [33].

### Immunohistochemical (IHC) staining

IHC staining of Ihh was performed using a Two-step Detection Kit from ZSGB-BIO (#PV-9001, Beijing, China). Briefly, cryosections of lower lumbar IVDs from 2-week-old mice were air-dried at RT, and incubated with 10 mM citric acid solution (pH 6.0) for 4 h at 60 °C for antigen retrieval. Subsequently, sections were blocked with endogenous peroxidase blocker (ZSGB-BIO), followed by incubation with Ihh primary antibody (1:100; Abcam; ab52919) for 60 min at 37 °C. Sections were incubated with reaction enhancement solution (ZSGB-BIO) for 60 min at RT and then incubated with enhanced enzyme-conjugated goat anti-rabbit IgG for 15 min at RT. After three washes with PBS, sections were stained with DAB reagent (ZLI-9019, ZSGB-BIO) and then counterstained with hematoxylin. Afterwards, sections were dehydrated with gradient alcohol, cleared with xylene, and finally sealed with neutral resin.

### X-gal and alkaline phosphatase (ALP) staining of IVD cryosections

To detect β-galactosidase (LacZ) activity in the IVD tissues, X-gal staining was performed on frozen sections as described previously [27, 29]. To assess activity of ALP, which is active during chondrocyte hypertrophy, ALP staining was performed as previously reported [27]. The stained sections in both experiments were examined and imaged with a Zeiss Axio Imager Z2 upright microscope (Carl Zeiss Microscopy).

### Immunofluorescence

Frozen sections of lower lumbar IVDs were subjected to antigen retrieval as described below. Specifically, the antigen retrieval for cytokeratin 19 (Krt19) was performed in 10 mM Tris-EDTA (pH 9.0) at 65 °C for 1 h. To retrieve antigens for Acan, ColX and Mmp13, IVD sections were digested with 2 mg/ml hyaluronidase (Sigma) at 55 °C for 2 h. After the antigen retrieval procedure, sections were blocked with 10% goat normal serum at RT for 1 h, and then incubated with primary antibodies for Acan (1:200, EMD Millipore, AB1031), Krt19 (1:200, Abcam, ab52625), ColX (1:1000, Abcam, ab58632), or Mmp13 (1:200, Abcam, ab39012) at 4 °C overnight. The next day, samples were stained with Alexa Fluor 647-labeled anti-rabbit secondary antibody (1:200, Invitrogen, A21246) at RT for 1 h, followed by counterstaining with DAPI for 5 min. Finally, samples were mounted with the M.W. mounting medium and then visualized under a fluorescence microscope. Quantitative analyses of immunofluorescent images were performed using the ImageJ software.

### RNA isolation and quantitative real time PCR (qPCR)

To isolate total RNA from IVD tissues, the lumbar IVDs from three 6-week-old mice were pooled, fully ground into a fine powder with a mortar in liquid nitrogen, and then extracted with the Column Cartilage RNAOUT kit (#101121, TIANDZ, Beijing, China) according to the manufacturer’s instructions. qPCR analysis was performed as described previously [34]. The relative expression level of each target gene was calculated by the 2^−ΔΔCT^ method using 18s ribosomal RNA as a loading control.

### Protein extraction and Western blot

Caudal IVDs from three 6-week-old mice of the same genotype were isolated, fully ground into a fine powder with a mortar in liquid nitrogen, and then extracted with radioimmunoprecipitation assay (RIPA) lysis buffer supplemented with protease and phosphatase inhibitors. Equal amounts of protein samples (15–30 µg) were separated by electrophoresis in 10% SDS-PAGE, and then transferred to 0.2 μm NC membrane (Amersham, 10600001). Subsequently, the blots were blocked with 5% skim milk in TBST at RT for 2 h, and then incubated with mouse primary antibodies against Smo (1:2000, #66851-1-Ig, Proteintech) or Gli1 (1:2000, #66905-1-Ig, Proteintech), goat primary antibodies against Gli2 (1:3000, #AF3635, R&D System) or Gli3 (1:2000, #AF3690, R&D System), or rabbit primary antibody against β-actin (1:2000, #4970S, Cell Signaling Technology) at 4 °C overnight with gentle shaking, followed by incubation with the corresponding HRP-labeled goat anti-mouse (1:4000, #D110087, BBI), rabbit anti-goat (1:4000, #D110117, BBI), or goat anti-rabbit (1:4000, #D110058, BBI) secondary antibodies RT for 1 h. After being washed 3 times with TBST, the blots were visualized using the chemiluminescent HRP Substrate (Millipore, WBKLS0500), and quantified using the Image J software.

### Statistics

Data were presented in dot-plots showing individual data points and the mean ± SD for each group. For quantitative analysis of µCT and histological data, each vertebral body or IVD was treated as an independent biological replicate. For qPCR or western blot analysis, lumbar discs or caudal discs from 3 mice with the same genotype were respectively collected, and considered as one independent sample. Statistical differences between two groups were evaluated by Student’s t-test. For comparisons among three or more groups, one-way or two-way ANOVA was performed, depending on whether one or two independent variables are involved. All statistical analyses were performed in PRISM 8 software (GraphPad Software, San Diego, CA, USA) and the difference was determined to be statistically significant when the resulting *p* value was below a threshold of 0.05.

## Results

### Hh-responsive cells are specifically localized in the inner AF and CEP, but not in the NP of postnatal IVDs

**Fig. 1:**
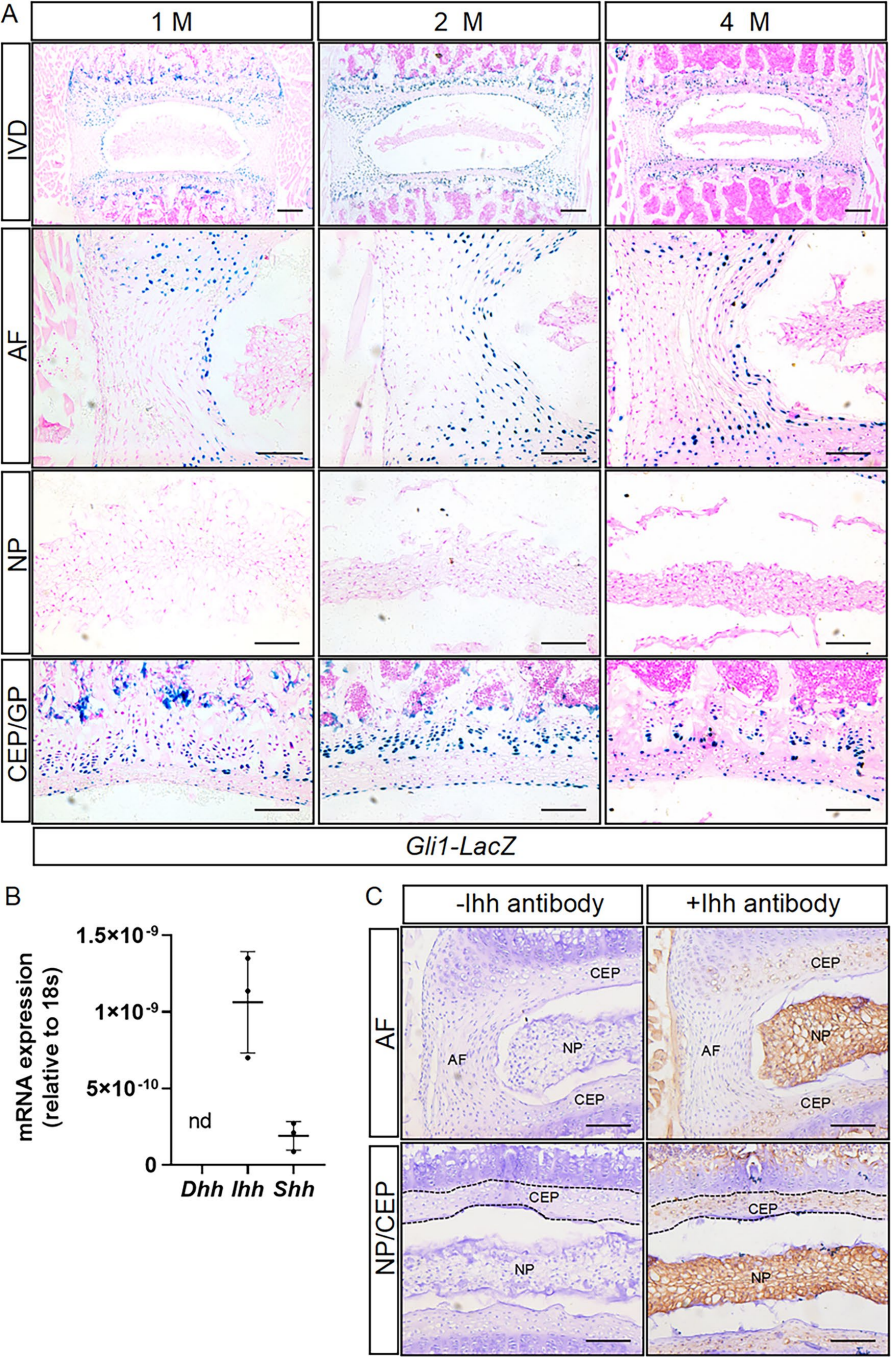
Hh-responsive cells are specifically localized in the inner annulus fibrosus and cartilaginous endplate, but not in the nucleus pulposus of postnatal IVDs. **A** Representative low- (top row) and high-magnification (other rows) images of X-gal staining in frozen sections of lumbar intervertebral discs (IVDs) from 1-, 2-, and 4-month-old *Gli1-LacZ* mice. LacZ-positive cells are stained blue. Scale bar: 200 μm in the top row of images; 100 μm in other images. **B** qPCR analysis of the mRNA levels of *Shh*, *Ihh*, and *Dhh* in the lumbar IVDs of 6-week-old mice. **C** Immunochemical (IHC) staining of Ihh in the paraffin sections of the lumbar IVDs from 2-week-old mice. Ihh-expressing cells were stained brown. Scale bar:100 μm. *IVD* intervertebral disc, *NP* nucleus pulposus, *AF* annulus fibrosus, *CEP* cartilaginous endplate, *GP* vertebral growth plate

To examine the distribution of Hh-responsive cells in postnatal IVDs, we utilized *Gli1-LacZ*, a *LacZ* knock-in allele of *Gli1* that is commonly used as a readout of Hh signaling activity and responsiveness [27, 29]. X-gal staining of longitudinal lumbar IVD sections from 1-, 2-, and 4-month-old *Gli1-LacZ* mice showed that IVDs exhibited the similar LacZ expression patterns at all these stages (Fig. 1A). Specifically, strong LacZ staining was observed in the cell types that were expected to exhibit high Hh activity, including chondrocytes of the vertebral growth plate (GP) as well as mesenchymal progenitors at the chondro-osseous junction below the GP (Fig. 1A). In addition, LacZ-positive cells were observed in AF and CEP, mainly localized in the inner AF and CEP, both of which are closely in contact with NP cells (Fig. 1A). However, no LacZ activity was detected in NP cells at all stages examined (Fig. 1A). Collectively, our results demonstrated that Hh signaling is highly active in the inner AF and CEP, but not in the NP compartment of postnatal IVDs.

Hh signaling can be activated by *Shh*, *Ihh* or *Dhh*. qPCR analysis showed that the mRNA level of *Ihh is* about 2.5-*fold* higher than that of *Shh*, whereas *Dhh* was nearly undetectable (Fig. 1B) in juvenile IVDs. IHC staining further confirmed that the Ihh protein is strongly expressed in the NP cells of 2-week-old IVDs (Fig. 1C, Supplemental Fig. 1). In addition, relatively weak signals were also detected in the CEP cells, but not in the AF cells (Fig. 1C, Supplemental Fig. 1). Thus, Ihh likely represents the major source of Hh ligand responsible for activating Hh signaling in the inner AF and CEP.

**Pharmacological inhibition of Hh signaling in juvenile mice causes decreased vertebral trabecular bone mass and early-onset spontaneous IVD degeneration**.

**Fig. 2:**
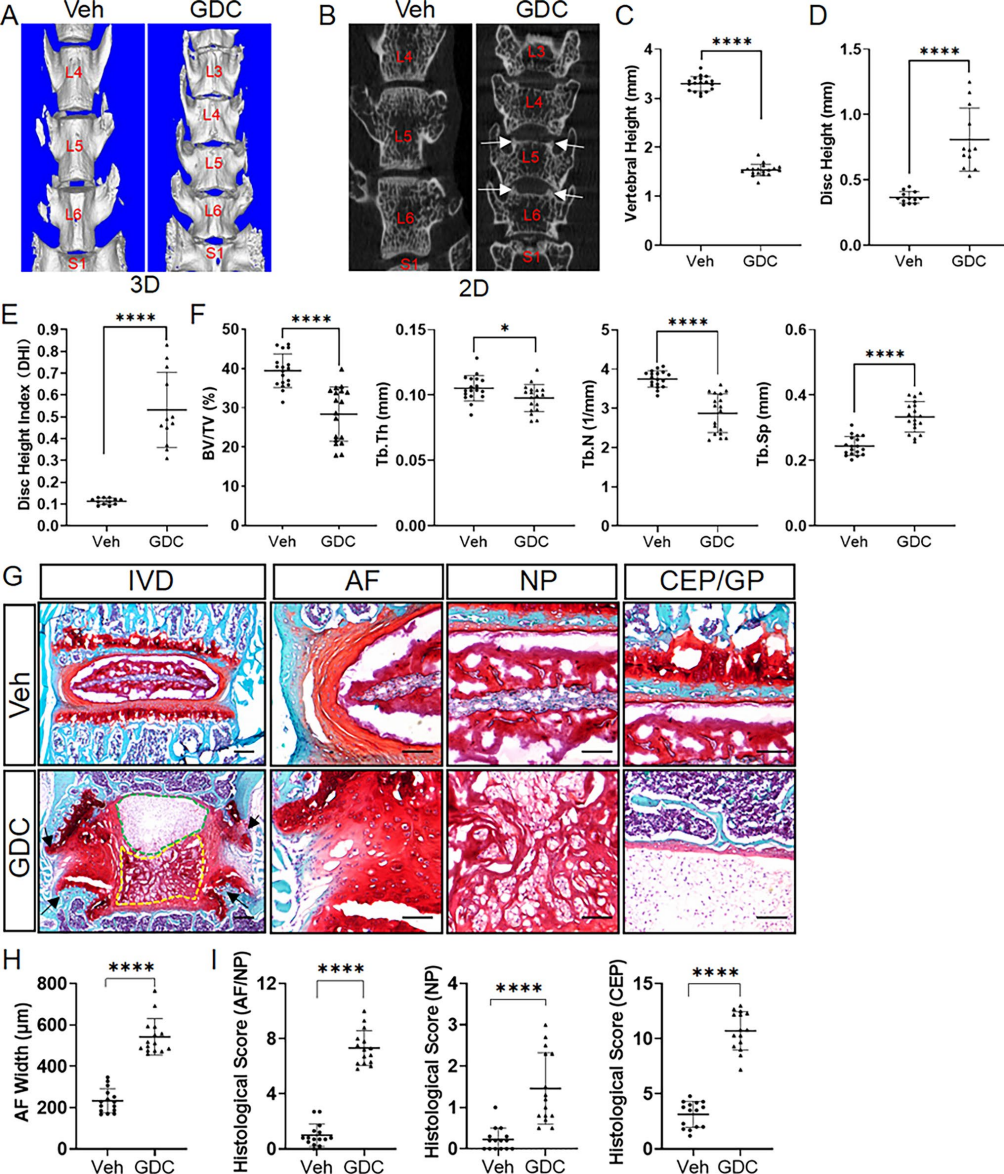
Pharmacological inhibition of Hh signaling in juvenile mice causes early-onset spontaneous degenerative changes in all disc compartments accompanied by decreased vertebral trabecular bone mass. **A**, **B** Representative 3D (**A**) and 2D (**B**) µCT images of lower lumbar spines from 4-month-old vehicle- (Veh) and GDC-treated mice. White arrows point to osteophytes. The vertebral levels are marked with red text. **C**–**E** Measurement of vertebral height (**C**), disc height (**D**), and disc height index (DHI) (**E**) from 2D µCT images of lower lumbar spines. n = 18 vertebrae (L4, L5, L6) or 12 IVDs (L4-L5, L5-L6) from 6 mice per group. **F** µCT quantification of the percentage of bone volume relative to total tissue volume (BV/TV), trabecular thickness (Tb.Th), trabecular number (Tb.N), and trabecular separation (Tb.Sp) in the trabecular regions of L4–6 vertebrae. n = 18 vertebrae (L4, L5, L6) from 6 mice per group. **G** Safranin O/Fast green staining of the mid-coronal sections of lower lumbar IVDs from 4-month-old vehicle- (Veh) and GDC-treated (GDC) mice. Black arrows point to osteophytes. Green or yellow dashed lines respectively enclose the immature NP region lacking the proteoglycan matrix or the degenerated NP region containing cell clusters isolated by the proteoglycan-rich matrix. Scale bar: 200 μm in low-magnification images, 100 μm in high-magnification images. **H** Measurement of AF width. **I** Histopathological scores for AF/NP, NP, and CEP for lower lumbar IVDs of 4-month-old vehicle- (Veh) and GDC-treated (GDC) mice. n = 15 IVDs (L4-L5, L5-L6, L6-S1) from 5 mice per group. All data were presented in dot-plots showing individual data points and their mean ± SD for each group. Each data point indicates a value from one vertebra or IVD. Statistical significance was determined using Student’s t-test. *p < 0.05; **p < 0.01; ***p < 0.001; ****p < 0.0001. *IVD* intervertebral disc, *NP* nucleus pulposus, *AF* annulus fibrosus, *CEP* cartilaginous endplate, *GP* vertebral growth plate

To test the physiological function of Hh signaling in spinal health, we next employed GDC to inhibit Hh signaling in juvenile mice. µCT analysis of lower lumbar spines showed that GDC treatment dramatically altered the gross morphology of the vertebral body and intervertebral disc (Fig. 2A, B). In addition, osteophyte, a pathological feature of IVD degeneration, was also observed in the spines of GDC-treated mice (Fig. 2B, white arrows). Quantitative analysis revealed an increase in disc height but a decrease in vertebral height, resulting in the increased disc height index (DHI) after GDC treatment (Fig. 2C–E). Furthermore, GDC-treated mice showed a significant reduction in vertebral trabecular bone mass (Fig. 2F). These results suggest that Hh signaling plays an important role in maintaining spinal morphology and vertebral trabecular bone mass.

To further examine the effect of GDC treatment on IVD integrity, Safranin-O/Fast Green staining was then performed on coronal sections of lower lumbar IVDs, which revealed the histological features of IVD degeneration in GDC-treated mice, such as the appearance of osteophytes (Fig. 2G, black arrows), discontinuity of the AF/NP boundary, reduced thickness of CEP, appearance of cell clusters isolated by the proteoglycan-rich extracellular matrix(ECM) in the NP tissues, and significantly expanded but severely disorganized AF (Fig. 2G, H). These degenerative changes led to higher NP/AF, NP, CEP histopathological scores for lower lumbar discs in GDC-treated mice in comparison with those in vehicle-treated mice (Fig. 2I). In addition, GDC treatment also resulted in an overall enlargement of the NP compartment and an obvious loss of vertebral growth plates (Fig. 2G). Taken together, these results suggest that Hh signaling is indispensable for postnatal IVD growth and homeostasis, and that its inactivation leads to early-onset spontaneous IVD degeneration associated with decreased vertebral trabecular bone mass.

### Pharmacological inhibition of Hh signaling in juvenile mice leads to abnormal disc cell differentiation and extracellular matrix deposition

**Fig. 3:**
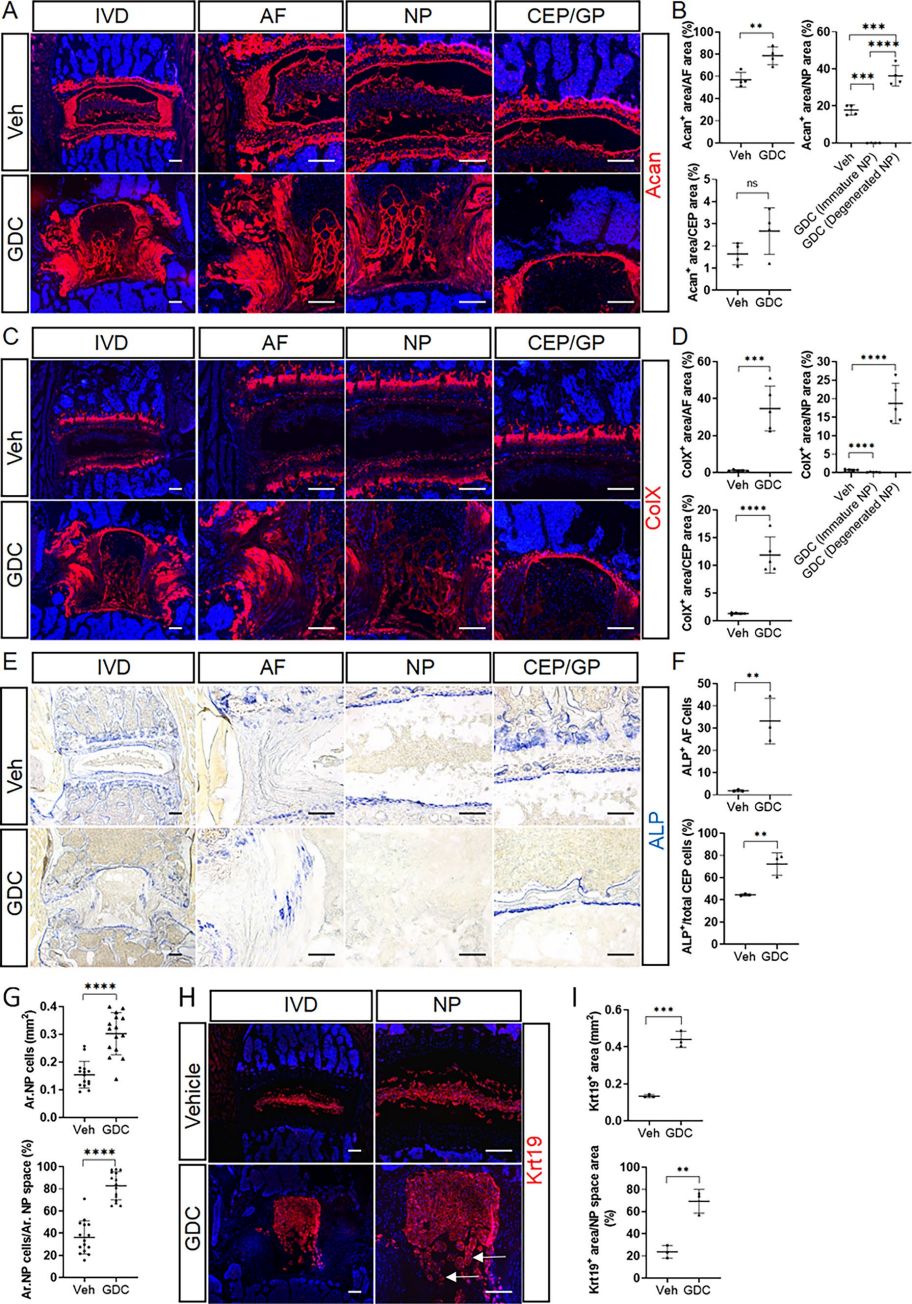
Pharmacological inhibition of Hh signaling in juvenile mice leads to abnormal disc cell differentiation and extracellular matrix deposition.  **A**–**D** immunofluorescent images (**A**, **C**) and quantitative analyses (B, D) of 4-month-old vehicle-(Veh) and GDC-treated lower lumbar IVDs stained with the antibodies against Acan (A, B) or ColX (C, D). n = 4–5 lower lumbar IVDs (one IVD/mouse) per group. **E** Alkaline phosphatase (ALP) staining was performed on cryosections of lower lumbar IVDs. **F** Quantitative analysis of ALP + cells in the AF and CEP compartment. n = 3 lower lumbar IVDs (one IVD/mouse) per group. **G** Measurement of the NP cell area (Ar. NP cells), and the percentage of NP cell area to total NP space area (Ar. NP cells/Ar. NP space) in the Safranin O/Fast green-stained lower lumbar IVD sections of 4-month-old vehicle- and GDC-treated mice. n = 15 IVDs (L4-L5, L5-L6, L6-S1) from 5 mice per genotype. **H** Immunofluorescent staining of 4-month-old lower lumbar discs with the anti-Krt19 antibody. White arrows point to Krt19-expressing NP cell clusters. **I** Quantitative analysis of the Krt19^+^ area and the percentage of Krt19^+^ cells relative to total NP space area. n = 3 lower lumbar IVDs (one IVD/mouse) per group. All data were presented in dot-plots showing individual data points and their mean ± SD for each group. Each data point indicates a value from one lower lumbar IVD. Statistical significance was determined using Student’s t-test or using one-way ANOVA with Tukey’s post-hoc test. *p  < 0.05; **p  < 0.01; ***p  < 0.001; ****p  < 0.0001. Scale bar: 200 μm in low-magnification images, 100 μm in high-magnification images. *IVD* intervertebral disc, *NP* nucleus pulposus, *AF* annulus fibrosus, *CEP* cartilaginous endplate, *GP* vertebral growth plate

The above histological changes in GDC-treated mice suggested abnormal disc cell phenotypes. Indeed, the AF of vehicle-treated mice contains fibroblast-like AF cells embedded in the concentric AF lamellae (Fig. 2G). In contrast, a large number of hypertrophic chondrocyte-like cells were found, and the typical lamellar structure was disrupted in GDC-treated AF (Fig. 2G), suggestive of abnormal differentiation of AF cells. Safranin O staining showed that the proteoglycan matrix was expressed specifically in the inner AF of control mice, whereas a poorly organized but significantly expanded proteoglycan matrix was observed in GDC-treated mice (Fig. 2G). Consistent with the results of histological analysis, quantitative immunofluorescence on lumbar discs showed that Acan, the extracellular matrix protein that is moderately expressed by inner AF, and ColX, the matrix protein that is highly expressed by hypertrophic chondrocytes and nearly absent in the healthy AF, were both significantly increased in the AF compartment of GDC-treated mice, but apparently disorganized (Fig. 3A–D). These matrix changes in GDC-treated discs were also associated with increased activity of ALP, which is known to be involved in chondrocyte hypertrophy and matrix mineralization, in the inner AF cells (Fig. 3E, F). Collectively, these results demonstrated that inhibition of Hh signaling leads to chondrogenic and hypertrophic differentiation of AF cells. Similarly, accelerated hypertrophic differentiation was also observed in the CEP of GDC-treated mice, as shown by the increased portion of ColX-expressing CEP area and higher percentage of ALP^+^ CEP cells relative to total CEP cells (Fig. 3C–F).

The effects of GDC treatment on NP cell differentiation were more complicated, since both decreased NP cell maturation and accelerated NP cell degeneration were observed in GDC-treated mice (Fig. 2G). NP cell differentiation during postnatal IVD maturation is characterized by a dramatic morphologic change from a vacuolated sphere to a spindle-like shape accompanied by the accumulation of extracellular proteoglycan around these cells [35]. However, these phenotypic changes in NP cells was nearly blocked by GDC treatment (Fig. 2G). Consistent with a defect in NP cell maturation, GDC-treated mice displayed a larger NP cell area (Ar. NP cells) and a higher percentage of NP cell area to total NP space area (Ar. NP cells/Ar. NP space) (Fig. 3G). Theses morphological results were further verified by quantitative immunofluorescence of Krt19, a specific marker of NP cells (Fig. 3H, I). Immunofluorescent analysis of NP further revealed that GDC treatment resulted in significantly reduced level of Acan protein in the immature NP region when mice reached 4 months of age (Fig. 3A–D). In addition to decreased NP cell maturation, accelerated NP cell degeneration was evident in some areas of GDC-treated NP (Fig. 2G) where cells were segregated into small clusters. Although these NP cell clusters still expressed NP-specific marker Krt19 (Fig. 3H, white arrows), they expressed high levels of Acan and ColX proteins (Fig. 3A, C), indicating the nature of chondrocytes. Collectively, these data indicate that pharmacological inhibition of Hh signaling leads to abnormal chondrogenic differentiation and extracellular matrix deposition of disc cells.

.

### Conditional deletion of *Smo* in disc cells of juvenile mice causes spontaneous AF and CEP degeneration accompanied by aberrant IVD cell phenotypes in adult mice

**Fig. 4:**
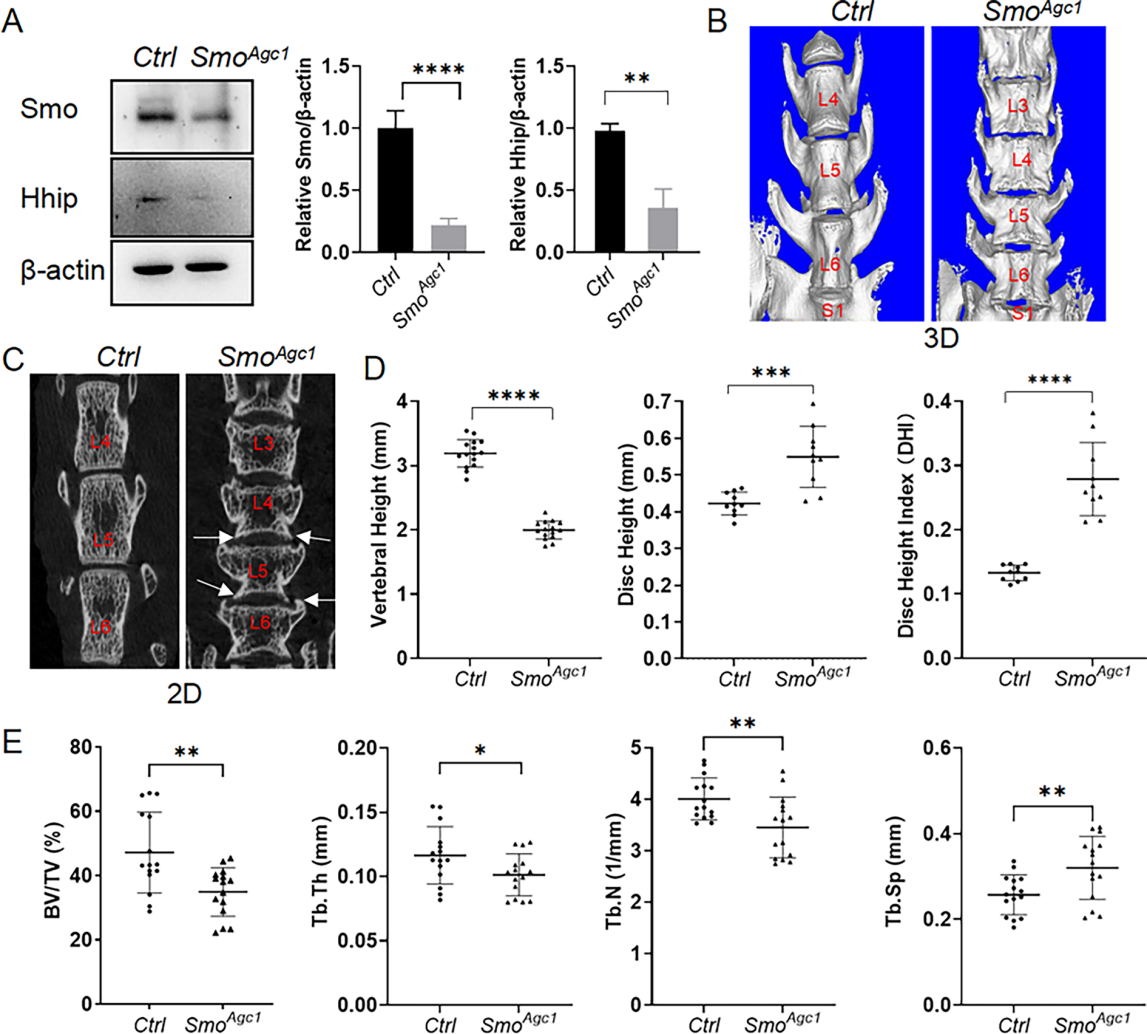
uCT analyses of the effect of *Agc1-CreER*^*T2*^*-*mediated deletion of *Smo* in juvenile mice on vertebral and intervertebral disc health. **A** Western blot analysis of caudal IVD tissues from 6-week-old *Smo*^*c/c*^ (*Ctrl*) and *Agc1-CreER*^*T2*^; *Smo*^*c/c*^ (*Smo*^*Agc1*^) mice. **B**–**C** Representative 3D (**B**) and 2D (**C**) µCT images of lower lumbar spines from 4-month-old *Smo*^*c/c*^ (*Ctrl*) and *Agc1-CreER*^*T2*^; *Smo*^*c/c*^ (*Smo*^*Agc1*^) mice. White arrows point to the osteophytes. **D** Determination of vertebral height, disc height, and disc height index (DHI) from 2D µCT images of the above mice. n = 15 vertebrae (L4, L5, L6) or 10 IVDs (L4-L5, L5-L6) from 5 mice per genotype. **E** µCT quantification of the percentage of trabecular bone volume relative to total tissue volume (BV/TV), trabecular thickness (Tb.Th), trabecular number (Tb.N), and trabecular separation (Tb.Sp) in the trabecular regions of L4–6 vertebrae from 4-month-old *Ctrl* and *Smo*^*Agc1*^ mice. n = 15 vertebrae (L4, L5, L6) from 5 mice per genotype. All mice *Ctrl* and *Smo*^*Agc1*^ were injected with tamoxifen once daily for 5 days starting at 2 weeks of age. Data were presented in dot-plots showing individual data points and their mean ± SD for each group. Each data point indicates a value from one vertebral body or IVD. Statistical significance was determined using Student’s t-test. *p < 0.05; **p < 0.01; ***p < 0.001; ****p < 0.0001

We next set to determine whether the IVD phenotypes in GDC-treated mice were caused by inactivation of Hh signaling in IVD cells. We and others have previously shown that the entire IVD cells in postnatal mice are targeted by *Agc1-CreER*^*T2*^, an inducible Cre driver in which a tamoxifen-inducible Cre recombinase was inserted into and therefore controlled by the endogenous aggrecan locus [3, 20], we therefore used *Agc1-CreER*^*T2*^ to conditionally ablate *Smo* in disc cells of juvenile mice. Specifically, *Agc1-CreER*^*T2*^; *Smo*^*c/c*^ mice (hereafter referred to as *Smo*^*Agc1*^) were generated by crossing mice carrying *Agc1-CreER*^*T2*^ allele with *Smo*^*c/c*^ mice, injected with tamoxifen at 2 weeks of age, and then assessed at different ages. Western blot analysis of IVD tissues showed a significant decrease in protein levels of Smo and Hhip (a target of Hh signaling) in 6-week-old *Smo*^*Agc1*^ mice compared with control mice (Fig. 4A), thus confirming effective ablation of *Smo* and subsequent inactivation of Hh signaling in these cells. µCT analyses showed that *Agc1-CreER*^*T2*^*-*mediated *Smo* deletion in 2-week-old mice resulted in the same vertebral and IVD phenotypes as GDC treatment by 4 months of age (Fig. 4B–E), suggesting that the function of Hh signaling in disc cells is critical for maintaining the vertebral and intervertebral disc health.

**Fig. 5:**
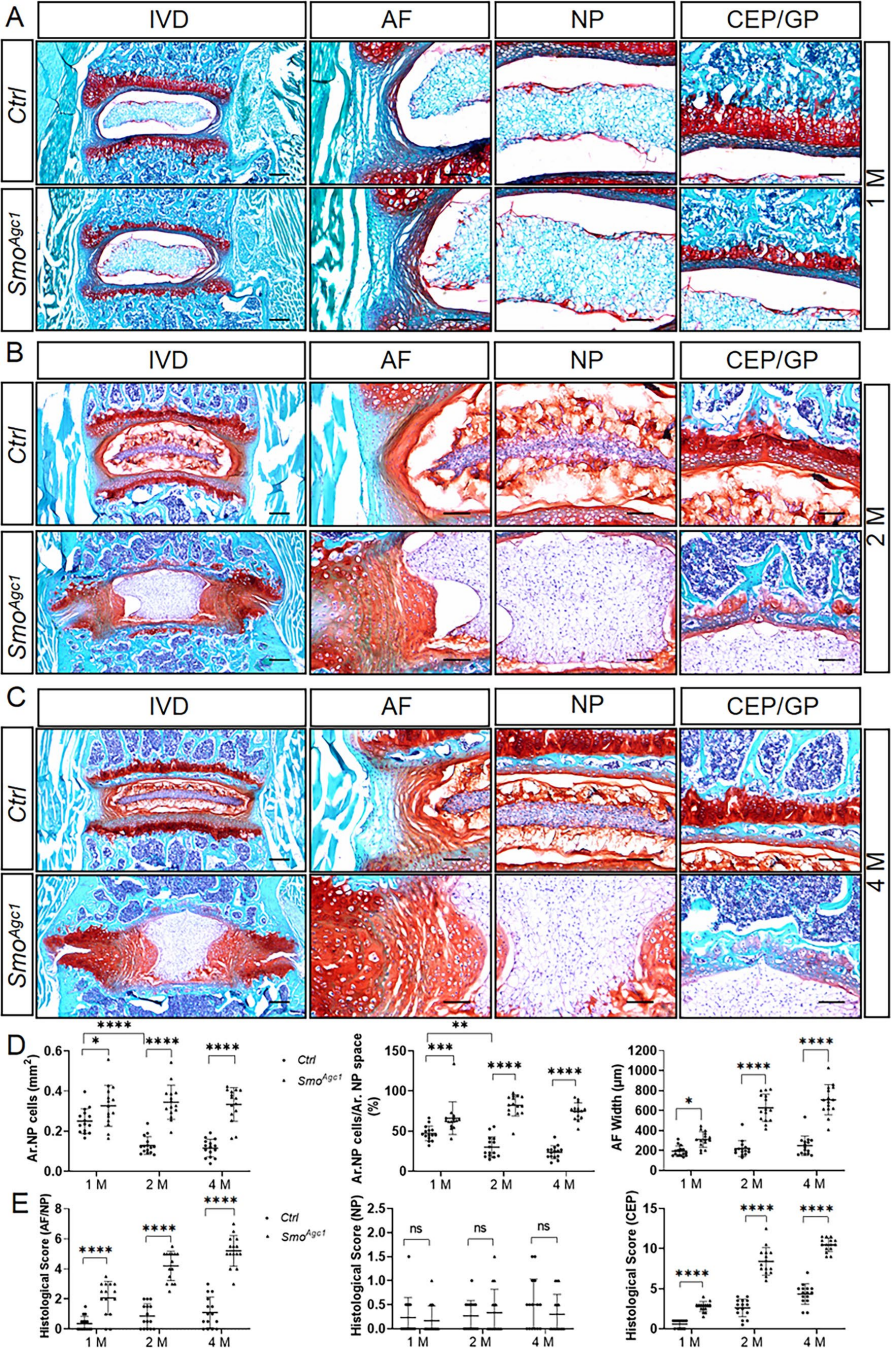
Histopathological analyses of the effect of *Agc1-CreER*^*T2*^*-*mediated deletion of *Smo* in juvenile mice on the lower lumbar IVDs. **A**–**C** Safranin O/Fast green staining of the mid-coronal sections of lower lumbar IVDs from 1- (**A**), 2- (**B**), and 4-month-old (**C**) *Smo*^*c/c*^ (*Ctrl*) and *Agc1-CreER*^*T2*^; *Smo*^*c/c*^ (*Smo*^*Agc1*^) mice. The leftmost panel of each row represents a low-magnification view of the entire IVD and the other panels are high-magnified images. IVD: intervertebral disc; NP: nucleus pulposus; AF: annulus fibrosus; CEP: cartilaginous endplate; GP: vertebral growth plate. **D** Quantitative analysis of the NP cell area (Ar. NP Cells), the percentage of NP cell area out of total NP space area (Ar. NP cells/Ar. NP space), and AF width in the above IVD sections. n = 15 IVDs (L4-L5, L5-L6, L6-S1) from 5 mice per genotype. **E** Evaluation of histopathological scores for AF/NP, NP, and CEP in lower lumbar IVDs of the above mice. n = 15 IVDs (L4-L5, L5-L6, L6-S1) from 5 mice per genotype. All above *Ctrl* and *Smo*^*Agc1*^ mice were injected with tamoxifen once daily for 5 days starting at 2 weeks of age. Scale bar: 200 μm in low-magnification images, 100 μm in high-magnification images. Data were presented in dot-plots showing individual data points and their mean ± SD for each group. Each data point indicates a value from one vertebral body or IVD. Statistical significance was determined using two-way ANOVA with Tukey’s multiple comparisons test. *p < 0.05; **p < 0.01; ***p < 0.001; ****p < 0.0001

To further understand the IVD phenotypes caused by *Smo* ablation, we performed Safranin O staining on lower lumbar IVD sections from 1-, 2-, and 4-month-old *Smo*^*Agc1*^ mice. The cell mass in the center of NP space of 1-month-old mutant mice moderately enlarged, but exhibited normal cell morphology (Fig. 5A). These mice also had slightly expanded AF, along with a small number of hypertrophic cells in the inner AF (Fig. 5A). At this stage, the early sign of degeneration was also observed in the CEP of mutant mice (Fig. 5A). These disc phenotypes were more pronounced at 2 and 4 months of age (Fig. 5B, C). Specifically, NP cells from 2- or 4-month-old control mice had been transformed into a flattened shape and compacted into a narrow central mass in the IVD space, which was surrounded by a matrix rich in proteoglycan, whereas the NP compartment of mutant mice at these two stages contained almost no proteoglycan-rich matrix. Instead, it was almost entirely occupied by large spherical cells, indicating a defect in the NP maturation. In addition to abnormal differentiation, the number of NP cells in mutant mice appeared to be significantly higher compared to their age-matched controls (Fig. 5B, C). The AF compartment of lumbar discs from 2 - or 4-month-old mutant mice recapitulated degenerative features observed in the GDC-treated AF. Similarly, the CEP in mutant mice exhibited significant cell loss and structural collapse at 2 months of age, which became more severe at 4 months of age. Consistent with the above morphological observations, quantitative analysis of lower lumbar IVD sections showed a progressive decline in the area of NP cells (Ar. NP cells) and the percentage of NP cell area relative to total NP space area (Ar. NP cells/Ar. NP space) between 1 and 4 months of age in control mice, but not in mutant mice (Fig. 5D). On the other hand, the AF width was relatively stable from 1, 2 to 4 months of age in control mice, but increased with age in *Smo*-deficient mice (Fig. 5D). These changes resulted in significantly higher histopathological scores for AF/NP and CEP, but a normal score for NP in mutant mice compared to controls (Fig. 5E). In addition to IVDs, *Smo* deficiency also led to partial loss of vertebral growth plate at 1 month of age and nearly complete loss by 4 months of age. Taken together, these results demonstrated that inactivation of Hh signaling in disc cells of juvenile mice leads to impaired NP maturation and progressive degeneration of AF and CEP accompanied by abnormal disc cell differentiation by 4 months of age.

### Conditional deletion of *Smo* in IVDs of juvenile mice causes changes in phenotypic markers of disc cells in adult mice

**Fig. 6:**
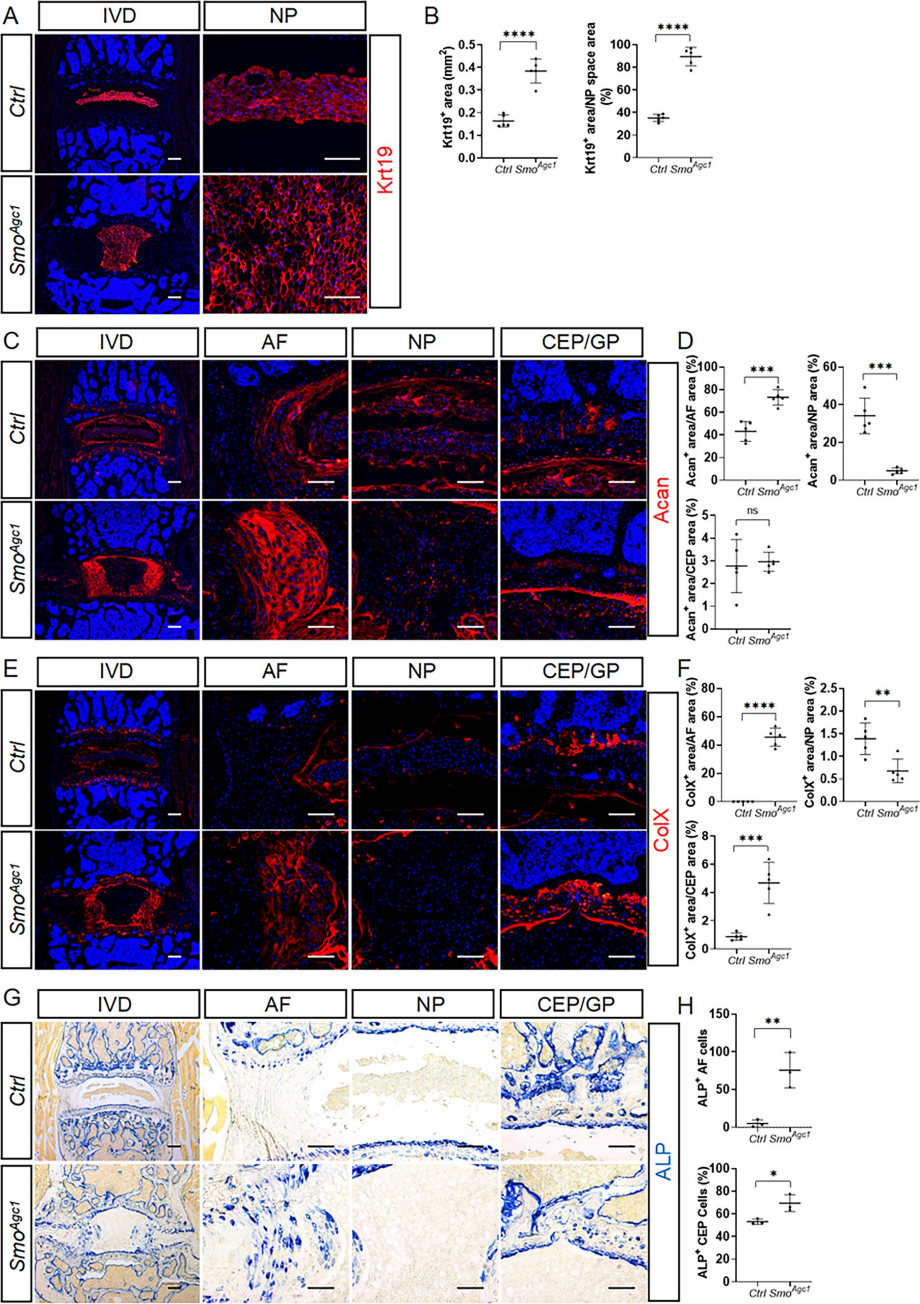
*Agc1-CreER*^*T2*^*-*mediated deletion of *Smo* in IVDs of juvenile mice causes changes in phenotypic markers of disc cells in adult mice. **A** Krt19 Immunofluorescent staining of lower lumbar IVDs from *Smo*^*c/c*^ (*Ctrl*) and *Agc1-CreER*^*T2*^; *Smo*^*c/c*^ (*Smo*^*Agc1*^) mice. **B** Quantitative analysis of the Krt19^+^ area and the percentage of Krt19^+^ area relative to total NP space area. n = 5. **C**–**F** immunofluorescent images (**C**, **E**) and quantitative analyses (**D**, **F**) of lower lumbar disc sections stained with the antibodies against Acan (**C**, **D**) and ColX (**E**, **F**). n = 5. **G** Alkaline phosphatase (ALP) staining of lower lumbar disc cryosections. **H** Quantitative analysis of ALP^+^ cells in the AF and CEP compartments. n = 3. All above *Ctrl* and *Smo*^*Agc1*^ mice were injected with tamoxifen once daily for 5 days starting at 2 weeks of age, and analyzed at 4 months of age. *IVD* intervertebral disc, *NP* nucleus pulposus, *AF* annulus fibrosus, *CEP* cartilaginous endplate, *GP* vertebral growth plate. Scale bar: 200 μm in low-magnification images, 100 μm in high-magnification images. Data were presented in dot-plots showing individual data points and their mean ± SD for each group. Each data point indicates a value from one lower lumbar IVD. Statistical significance was determined using Student’s t-test. *p < 0.05; **p < 0.01; ***p < 0.001; ****p < 0.0001

To gain further insights into the effect of *Smo* ablation on disc cell differentiation, we next examined the abundance and distribution of phenotypic markers of disc cells in lower lumbar IVDs of 4-month-old *Ctrl* and *Smo*^*Agcl*^ mutant mice. Quantitative immunofluorescence of Krt19 revealed that nearly all NP cells expressed Krt19 in similar abundance in both control and mutant IVDs (Fig. 6A). However, Krt19^+^ NP cells in mutant discs exhibited the morphology of immature NP cells (Fig. 6A), and their total and relative areas were significantly larger than those of control discs (Fig. 6B). Furthermore, both Acan and ColX were significantly downregulated in the NP tissues of *Smo* mutant mice compared to controls (Fig. 6C–F), suggesting that ablation of *Smo* in mice at 2 weeks of age disrupted NP cell differentiation associated with NP maturation, but did not cause NP degeneration by 4 months of age. Abnormal differentiation of AF cells was also confirmed by dysregulated expression of their phenotypic markers. The moderate level of Acan was restricted to the inner AF of control mice (Fig. 6C, D). However, its abundance is notably increased and its localization was remarkedly expanded in the AF of mutant mice. Similarly, ColX was nearly undetectable in the control AF, but abundantly expressed in the AF of *Smo* mutant mice (Fig. 6E, F). Furthermore, *Smo* deletion led to a significant increase in the number of cells exhibiting ALP activity in the inner AF (Fig. 6G, H). In contrast, it had no notable effect on the expression of Mmp13, a specific marker of terminally differentiated chondrocytes, in the AF (Supplemental Fig. 2). Thus, AF cells in mutant mice appeared to undergo chondrogenic and hypertrophic differentiation, but did not reach the terminally differentiated stage. Furthermore, similar to GDC treatment, *Smo* depletion resulted in a significant increase in the proportion of ColX^+^ area (Fig. 6E, F) and an increase in the percentage of cells showing ALP activity in the CEP (Fig. 6G, H). Thus, *Agc1-CreER*^*T2*^*-*mediated deletion of *Smo* in disc cells of juvenile mice caused changes in disc cell phenotypic markers, further suggesting that Hh signaling functions in the IVDs to promote NP cell maturation, but restrain aberrant chondrogenic differentiation of AF and CEP cells.

### Genetic deletion of *Smo* specifically in NP cells of juvenile mice leads to healthy IVD and normal disc cell differentiation in adult mice

**Fig. 7:**
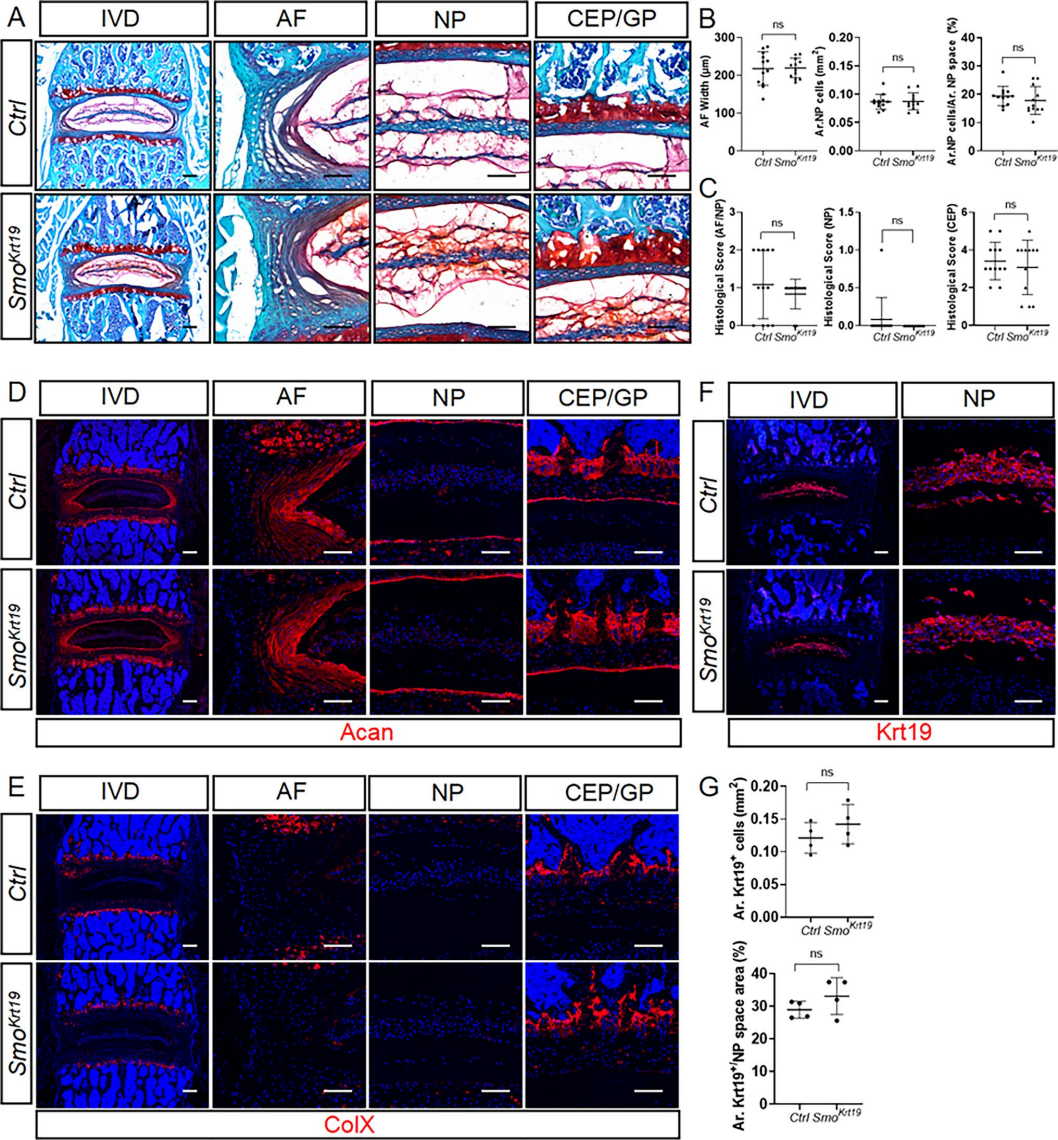
*Krt19-CreER*-mediated deletion of *Smo* in NP cells of juvenile mice leads to healthy IVD and normal disc cell differentiation in adult mice. **A** Safranin O/Fast green staining of the mid-coronal sections of lower lumbar IVDs from *Smo*^*c/c*^ (*Ctrl*) and *Krt19-CreER; Smo*^*c/c*^ (*Smo*^*Krt19*^) mice. The left panel in each row is low-magnification images of the entire IVD and the other panels are high-magnified views. **B** Quantitative analysis of the NP cell area (Ar. NP Cells), the percentage of NP cell area to total NP space area (Ar. NP cells/Ar. NP space), and AF width in the lower lumbar IVD sections of *Ctrl* and *Smo*^*Krt19*^ mice. n = 12 IVDs (L4-L5, L5-L6, L6-S1) from 4 mice per genotype. **C** Evaluation of histopathological scores for AF/NP, NP, and CEP in lower lumbar IVDs of the above mice. n = 12 IVDs (L4-L5, L5-L6, L6-S1) from 4 mice per genotype. (D-E) Representative immunofluorescent images of lower lumbar disc sections stained with the antibodies against Acan (**D**) and ColX (**E**). **F**, **G** Immunofluorescent images (**F**) and quantitative analyses (**G**) of Krt19 staining of lower lumbar disc sections from *Ctrl* and *Smo*^*Krt19*^ mice. n = 4. All above mice were injected with tamoxifen once daily for 5 days starting at 2 weeks of age and analyzed at 4 months of age. *IVD* intervertebral disc, *NP* nucleus pulposus, *AF* annulus fibrosus, *CEP* cartilaginous endplate, *GP* vertebral growth plate. Scale bar: 200 μm in low-magnification images, 100 μm in high-magnification images. Data were presented in dot-plots showing individual data points and their mean ± SD for each group. Each data point indicates a value from one lower lumbar IVD. Statistical significance was determined using Student’s t-test. *p < 0.05; **p < 0.01; ***p < 0.001; ****p < 0.0001

Next, we further investigated whether the above disc phenotypes are caused by disruption of Hh signaling in NP cells. We utilized tamoxifen-inducible *Krt19-CreER*, a Cre mouse line that has been shown to specifically target the entire NP cells in postnatal IVDs [36]. By employing *R26-tdTomato* fluorescent Cre reporter, we first validated that *Krt19-CreER* can specifically and effectively target NP cells when induced with tamoxifen at 2 weeks of ages (Supplemental Fig. 3). We then injected tamoxifen into *Krt19-CreER; Smo*^*c/c*^ (hereinafter *Smo*^*Krt19*^) and their littermate control mice at 2 weeks of ages and analyzed them at 4 months of age. Safranin O staining of lower lumbar IVDs revealed that NP-specific deletion of *Smo* have no significant impact on the AF, NP, and CEP compartments (Fig. 7A–C). Consistent with these histological results, immunofluorescence analysis of lower lumbar IVD sections from *Smo*^*Krt19*^ mice further revealed similar distribution and abundance of Acan (Fig. 7D), and ColX (Fig. 7E), Krt19 (Fig. 7F, G) between control and mutant discs. Taken together, these results demonstrated that the function of Hh signaling in NP cells is not required for IVD health and disc cell differentiation.

### Hh signaling functions in the AF/CEP tissues to indirectly prevent NP degeneration during aging

**Fig. 8:**
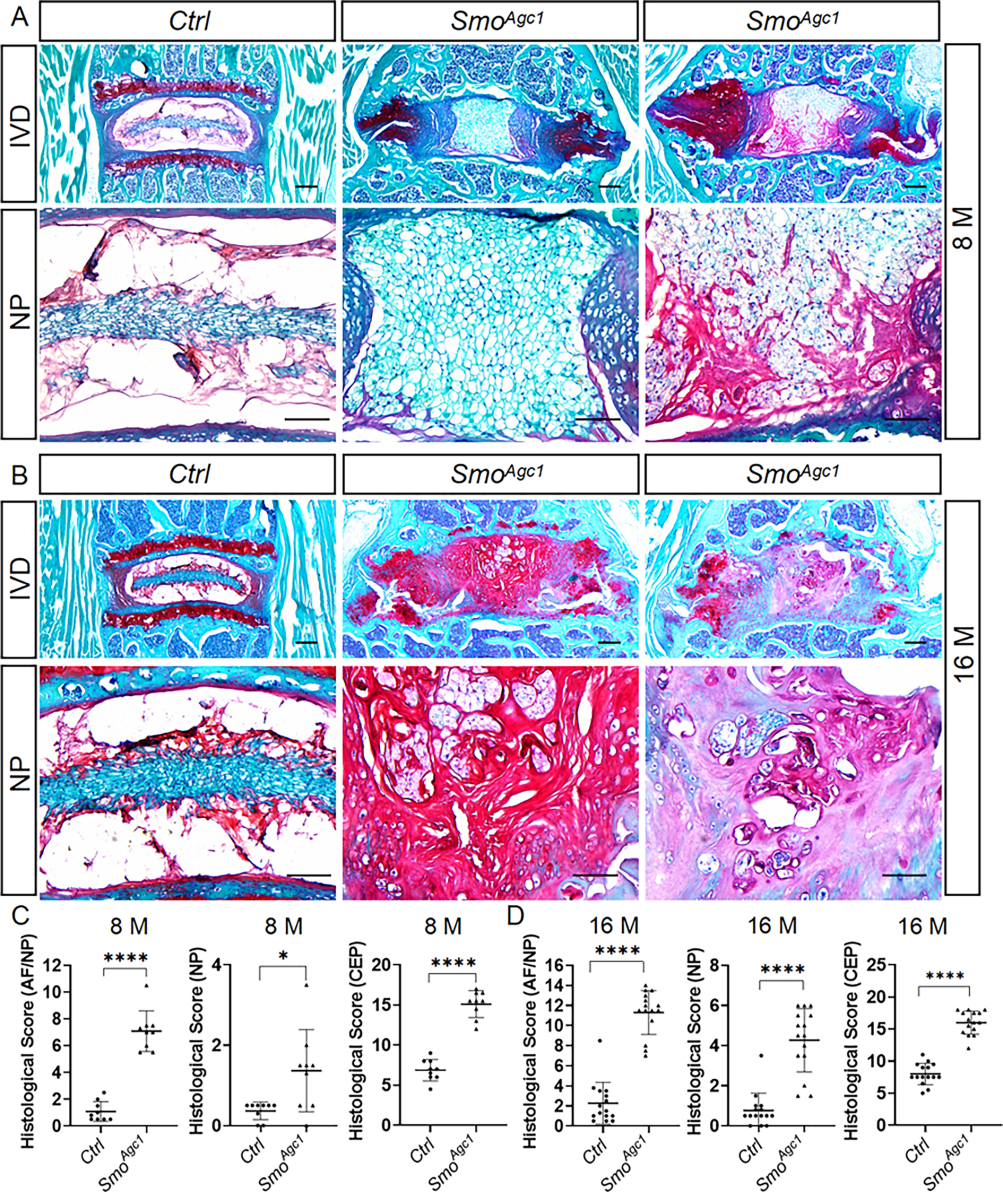
Inactivation of Hh signaling in the AF/CEP tissues indirectly accelerates NP degeneration during aging. **A**, **B** Safranin O/Fast green staining of the mid-coronal sections of lumbar IVDs from 8- (**A**) and 16-month-old (**B**) *Smo*^*c/c*^ (*Ctrl*) and *Agc1-CreER*^*T2*^; *Smo*^*c/c*^ (*Smo*^*Agc1*^) mice. Images in the top row: low-magnification of the entire IVDs; Other images: high-magnification view. *IVD* intervertebral disc, *NP* nucleus pulposus, *AF* annulus fibrosus, *CEP* cartilaginous endplate, *GP* vertebral growth plate. Scale bar: 200 μm in low-magnification images, 100 μm in high-magnification images. **C**, **D** Evaluation of histopathological scores for AF/NP, NP, and CEP in lower lumbar IVDs from 8- (**C**) and 16-month-old mice. All above *Ctrl* and *Smo*^*Agc1*^ mice were injected with tamoxifen once daily for 5 days starting at 2 weeks of age. n = 9 (8-month-old) or 15 (16-month-old) discs per genotype. Data were presented in dot-plots showing individual data points and their mean ± SD for each group. Each data point indicates a value from one lower lumbar IVD. Statistical significance was determined using Student’s t-test. *p < 0.05; **p < 0.01; ***p < 0.001; ****p < 0.0001

The above results have clearly showed that *Smo* deletion in disc cells led to early-onset degenerative changes in AF and CEP tissues, but not NP tissue in mice up to 4 months of age. We then asked whether these AF/CEP defects caused by *Smo* ablation affect NP degeneration during aging. To address this question, Safranin O staining was performed on lower lumbar IVD sections from 8- and 16-month-old *Smo*^*Agc1*^ mice. At 8 months of age, the NP tissues in nearly all mutant mice were still predominantly composed of large vacuolated cells (Fig. 8A). However, some mutant mice showed early signs of NP degeneration, such as discontinuity of NP/AF boundary and appearance of nested NP cells (Fig. 8A). At 16 months of ages, *Smo*^*Agc1*^mutant mice displayed varying degrees of degenerative changes in the NP tissues and most of them exhibited histological features typical of age-related disc degeneration, including matrix-rich NP compartment with reduced number of NP cells, loss of NP/AF boundary, and appearance of chondrocyte-like cells throughout the NP compartment (Fig. 8B). In some severe cases, the mutant NP tissues exhibited severe clefts and contained little cells (Fig. 8B, right panels). As a result, the NP histopathological scores were mildly increased at 8 months of age, but further worsened at 16 months of age in lower lumbar IVDs of mutant mice compared to those of age-matched control mice (Fig. 8C, D). In addition, the structural abnormalities of AF were also more severe in 8- and 16-month-old mutant mice than their younger counterparts, as evidenced by moderate to severe clefts and completely disrupted lamellar structure (Fig. 8A, B). Similarly, the CEP and vertebral growth plate were almost completely destroyed at these two stages (Fig. 8A, B). Analyses of histopathological scores for AF/NP and CEP in control and mutant mice further confirmed the above observations (Fig. 8C, D). Thus, *Agc1-CreER*^*T2*^*-*mediated deletion of *Smo* in disc cells accelerated age-related NP degeneration. In contrast, NP-specific deletion of *Smo* in juvenile mice have no discernible effect on age-related IVD degeneration (Supplemental Fig. 4). Taken together, these results demonstrated that Hh signaling plays a non-cell-autonomous role in maintaining the NP cell phenotypes, and that its inactivation indirectly leads to accelerated NP degeneration during aging.

### Hh signaling regulates IVD growth and homeostasis primarily by antagonizing Gli3 repressor

**Fig. 9:**
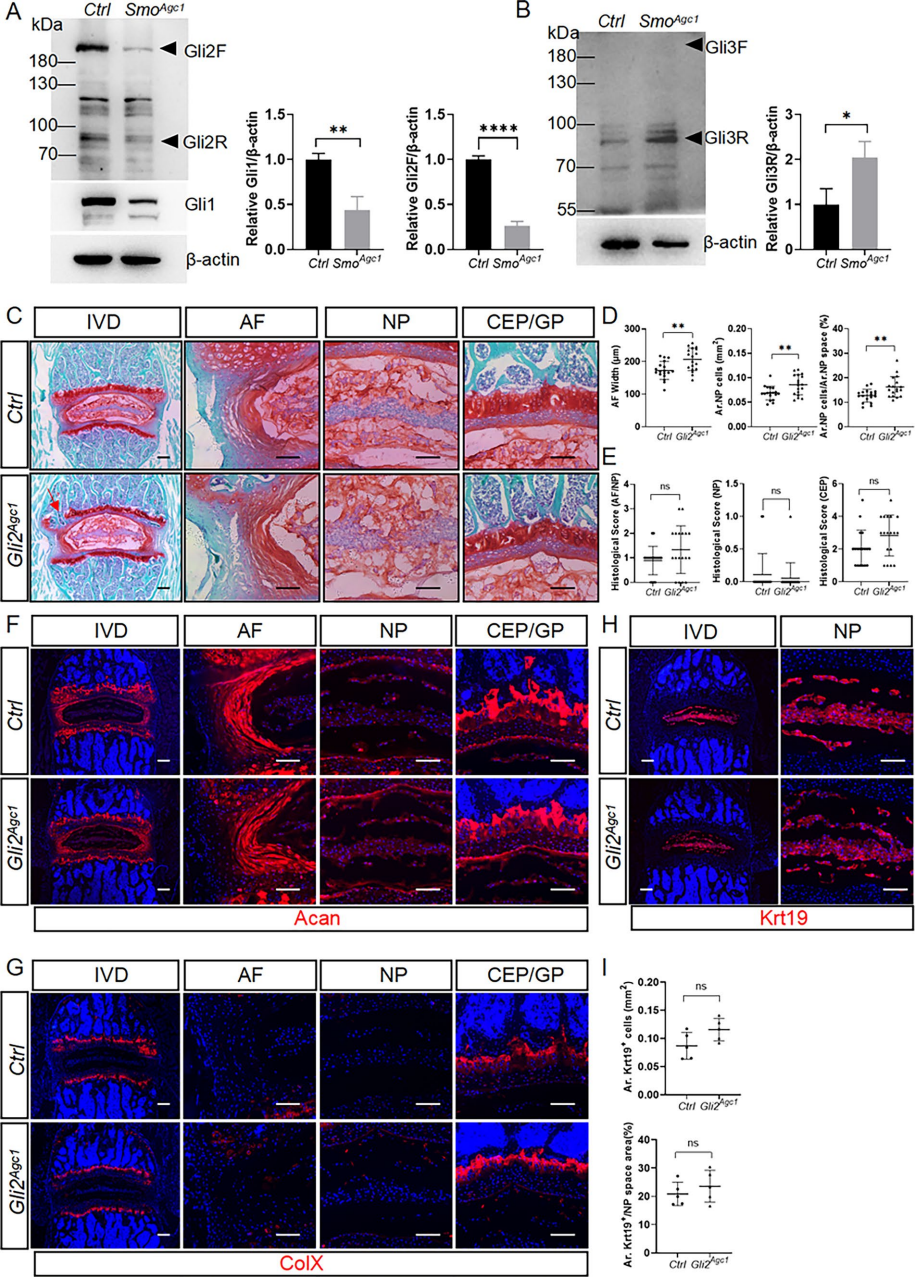
The function of Hh signaling in IVD cells is not primarily mediated by Gli2 activator during IVD growth. **A**, **B** Western blot analysis of Gli1, Gli2, (**A**) and Gli3 (**B**) proteins in IVD tissues from 6-week-old *Smo*^*c/c*^ (*Ctrl*) and *Agc1-CreER*^*T2*^; *Smo*^*c/c*^ (*Smo*^*Agc1*^) mice. Gli2F: full length Gli2 activator; Gli2R: truncated Gli2 repressor; Gli3F: full length Gli3 activator; Gli3R: truncated Gli3 repressor. **C** Safranin O/Fast green staining of the mid-coronal sections of lower lumbar IVDs from 4-month-old *Gli2*^*flox/flox*^ (*Ctrl*) and *Agc1-CreER*^*T2*^; *Gli2*^*flox/flox*^ (*Gli2*^*Agc1*^) mice. The left panel in each row is low-magnification images of the entire IVD and the other panels are high-magnified views. Red arrow points to prematurely fused growth plate. **D** Quantitative analysis of the NP cell area (Ar. NP Cells), the percentage of NP cell area to total NP space area (Ar. NP cells/Ar. NP space), and AF width in the lower lumbar IVD sections of *Ctrl* and *Gli2*^*Agc1*^ mice. n = 18 IVDs (L4-L5, L5-L6, L6-S1) from 6 mice per genotype. **E** Evaluation of histopathological scores for AF/NP, NP, and CEP in lower lumbar IVDs of the above mice. n = 18 IVDs (L4-L5, L5-L6, L6-S1) from 6 mice per genotype. **F**, **G** Representative immunofluorescent images of lower lumbar disc sections stained with the antibodies against Acan (**F**) and ColX (**G**). **H**, **I** Immunofluorescent images (**H**) and quantitative analyses (**I**) of Krt19 staining of lower lumbar disc sections from *Ctrl* and *Gli2*^*Agc1*^ mice. n = 5. All above mice were injected with tamoxifen once daily for 5 days starting at 2 weeks of age. *IVD* intervertebral disc, *NP* nucleus pulposus, *AF* annulus fibrosus, *CEP* cartilaginous endplate, *GP* vertebral growth plate. Scale bar: 200 μm in low-magnification images, 100 μm in high-magnification images. Data were presented in dot-plots showing individual data points and their mean ± SD for each group. Each data point indicates a value from one IVD. Statistical significance was determined using Student’s t-test. *p < 0.05; **p < 0.01; ***p < 0.001; ****p < 0.0001

The biological functions of Hh signaling are mediated by Gli family of transcriptional factors. To investigate the effect of *Smo* deletion on Gli factors, western blot analysis was performed on protein extracts of IVD tissues from 6-week-old mice. The results showed that Gli2 and Gli3 were mainly present as GliA and GliR in the control IVD tissues, respectively, and that the protein levels of Gli1 and Gli2A were significantly reduced, while Gli3R was significantly increased in the IVD tissues of *Smo*^*Agcl*^ mice, compared to those of control mice (Fig. 9A, B). To determine the potential roles of GliA in mediating Hh signaling function in IVDs, we used *Agc1-CreER*^*T2*^ to conditionally delete *Gli2*, the major *Gli* transcriptional activator, from IVD cells. We reasoned that if Gli2A was the primary effector of Hh signaling in postnatal IVDs, *Gli2* mutant mice would exhibit the similar IVD phenotypes to *Smo*^*Agcl*^ mice. Safranin O staining of lower lumbar IVDs revealed that *Agc1-CreER*^*T2*^; *Gli2*^*flox/flox*^ (hereinafter *Gli2*^*Agc1*^) mice had overall normal IVD morphology except that vertebral growth plates in some of these mice were partially lost (Fig. 9C, red arrow). Morphometric analysis showed that *Gli2*^*Agc1*^ mice had subtle increases in AF width, Ar. NP cells, and Ar. NP cells/Ar.NP space area, but did not show obvious degenerative changes (Fig. 9D). Consistent with these observations, histopathological scores of NP, AF/NP, and CEP in *Gli2* knockout mice were not statistically different from those in control mice, although the CEP score in *Gli2*^*Agc1*^ mice did tend to increase compared with *Ctrl* mice (Fig. 9E). Furthermore, quantitative immunofluorescence analysis of lower lumbar IVD sections showed largely normal expression of Acan (Fig. 9F), ColX (Fig. 8G), and Krt19 (Fig. 9H, I) in the IVDs of *Gli2*^*Agc1*^ mutant mice compared with control mice. Taken together, these results suggested that although Gli2A is important for maintenance of vertebral growth plate, it plays a minor role in mediating Hh signaling in discs cells during IVD growth.

**Fig. 10:**
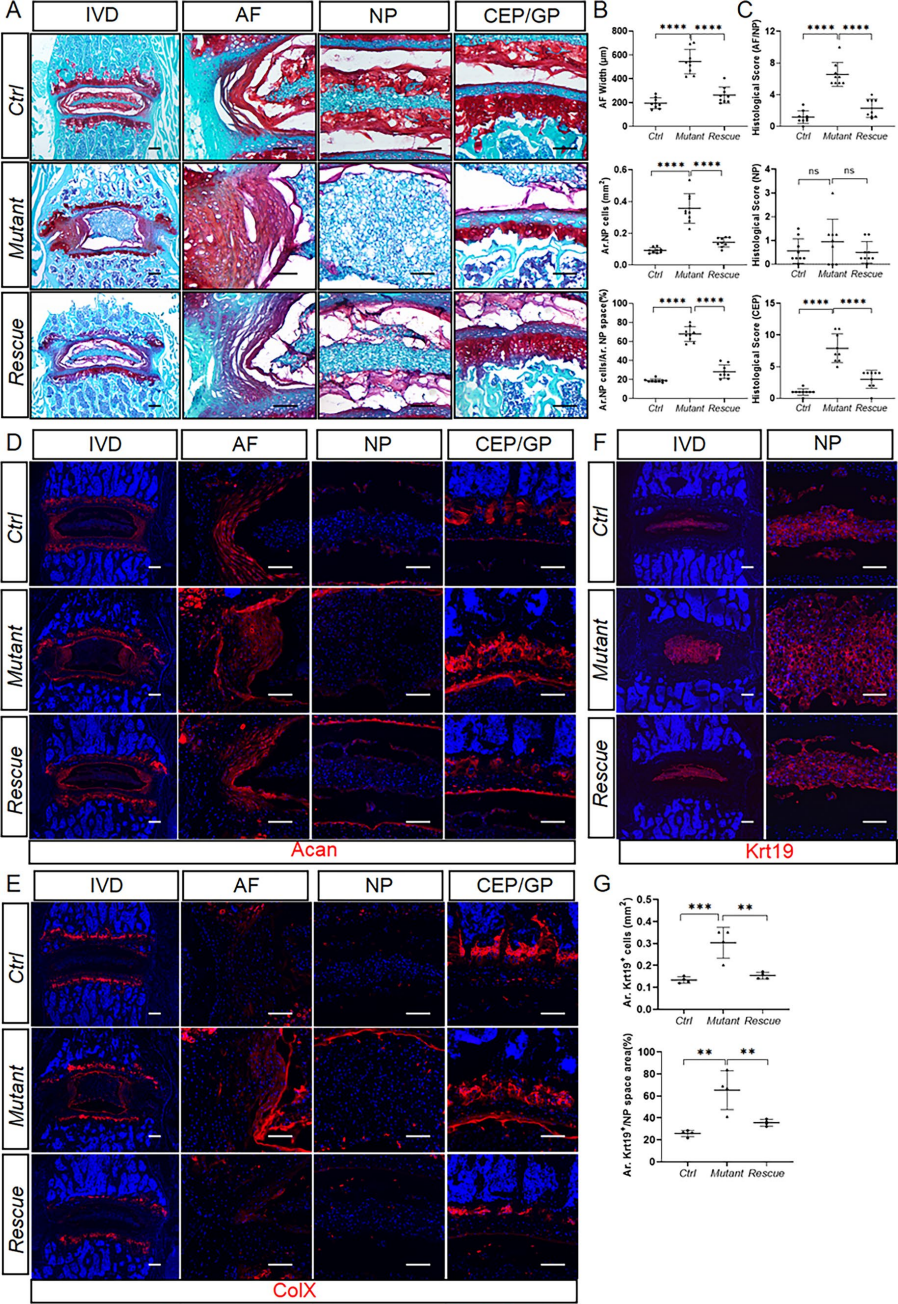
Hh signaling regulates IVD growth and homeostasis primarily by antagonizing Gli3 repressor. **A** Safranin O/Fast green staining of the mid-coronal sections of lower lumbar IVDs from 4-month-old *Smo*^*c/c*^ (*Ctrl*), *Agc1-CreER*^*T2*^; *Smo*^*c/c*^; *Gli3*^*flox/+*^(*Mutant*), and *Agc1-CreER*^*T2*^; *Smo*^*c/c*^; *Gli3*^*flox/flox*^ (*Rescue*) mice. **B** Quantitative analysis of the NP cell area (Ar. NP Cells), the percentage of NP cell area to total NP space area (Ar. NP cells/Ar. NP space), and AF width in lower lumbar IVD sections of the above mice. in the lower lumbar IVD sections of *Ctrl*, *Mutant*, and *Rescue* mice. n = 9 discs per genotype. **C** Evaluation of histopathological scores for AF/NP, NP, and CEP in lower lumbar IVDs of the above mice. n = 9 discs per genotype. **D**, **E** Representative immunofluorescent images of lower lumbar disc sections stained with the antibodies against Acan (**D**) and ColX (**E**). **F**, **G** Immunofluorescent images (**F**) and quantitative analyses (**G**) of Krt19 staining of lower lumbar disc sections of indicated groups. n = 4 discs per genotype. All above mice were injected with tamoxifen once daily for 5 days starting at 2 weeks of age and analyzed at 4 months of age. *IVD* intervertebral disc, *NP* nucleus pulposus, *AF* annulus fibrosus, *CEP* cartilaginous endplate, *GP* vertebral growth plate. The left panel in each row is low-magnification images of the entire IVD and the other panels are high-magnified view. Scale bar: 200 μm in low-magnification images, 100 μm in high-magnification images. Data were presented in dot-plots showing individual data points and their mean ± SD for each group. Each data point indicates a value from one IVD. Statistical significance was determined using one-way ANOVA with Tukey’s post-hoc test. *p < 0.05; **p < 0.01; ***p < 0.001; ****p < 0.0001

We next evaluated the potential contribution of Gli3R to the function of Hh signaling in IVDs. To this end, we generated *Agc1-CreER*^*T2*^; *Smo*^*c/c*^; *Gli3*^*flox/+*^allele (*Mutant*) and *Agc1-CreER*^*T2*^; *Smo*^*c/c*^; *Gli3*^*flox/flox*^ (*Rescue*) mice to conditionally delete one and both alleles of *Gli3* in *Agc1-CreER*^*T2*^; *Smo*^*c/c*^ background at 2 weeks of age, respectively. Safranin O staining of lower lumbar IVD sections revealed that 4-month-old *Mutant* mice exhibited the similar defects in AF/CEP degeneration and NP maturation to age-matched *Smo*^*Agc1*^ mice (Fig. 10A). Interestingly, the aforementioned defects were significantly attenuated in *Rescue* mice (Fig. 10A). Consistently, AF width, Ar.NP cells, and Ar.NP cells/Ar. NP space in lower lumbar IVDs were significantly smaller in *Rescue* mice than in *Mutant* mice (Fig. 10B). Similarly, the histopathological scores for AF/NP and CEP were notably decreased by the loss of two alleles of *Gli3* (Fig. 10C). Consistent with these histological results, immunofluorescence analysis of lower lumbar IVD sections further revealed that simultaneous deletion of both alleles of *Gli3* largely reversed abnormal distribution and abundance of Acan (Fig. 10D), ColX (Fig. 10E), and Krt19 (Fig. 10F, G) caused by *Smo* deletion in the IVDs. Taken together, these results demonstrated that Hh signaling regulates IVD growth and homeostasis primarily by antagonizing Gli3R.

## Discussion

IVD cells, in particular NP or AF cells, undergo phenotypic changes in response to certain pathological conditions such as overload or aging, and therefore result in aberrant composition and organization of extracellular matrix, which in turn undermines the structural and biomechanical properties of IVDs and ultimately causes clinical symptoms such as low back pain [2]. During the past decades, some progresses have been made in revealing molecular regulators of NP and AF cells during postnatal growth and homeostasis [4–6, 8, 9, 31]. However, despite these efforts, information on signaling pathways that regulate disc cell phenotypes remains limited. In this study, we utilized a Smo inhibitor and multiple genetic tools to dissect the physiological role of Hh signaling in postnatal IVDs. We found that either global inhibition of Hh signaling by GDC or disc-specific inactivation of Hh signaling in juvenile mice caused profound changes in the AF/CEP and NP tissues by adulthood. In addition, our data also demonstrated that genetic inactivation of Hh signaling exacerbated age-related IDD. These results clearly indicate that Hh signaling plays a critical role in the IVD growth and homeostasis, and its inactivation is associated with IVD degeneration during aging. Mechanistically, we showed that Hh signaling functions in IVDs mainly by antagonizing the Gli3 repressor. Overall, our findings provide new insights into the complex role of Hh signaling in IVD homeostasis and degeneration, and suggest that targeted activation of Hh signaling may represent a potential therapeutic strategy for the prevention and treatment of IDD.

Both genetic ablation of *Shh* or *Ihh* in neonate mice and pharmacological inhibition of Smo in 8-week-old mice led to mild defects in AF and CEP [14, 16, 17], suggesting that Hh signaling may regulate AF/CEP growth and homeostasis at these stages. However, these studies could not distinguish between the direct requirement of Hh signaling in AF/CEP cells and its indirect effect on these cells. To explicitly address this question, we here used *Gli1-LacZ* reporter mice to show that Hh signaling is activated in the inner AF and CEP cells of juvenile and adult mice. Moreover, we showed that *Smo* loss in disc cells of juvenile mice leads to early-onset AF and CEP degeneration associated with abnormal cell differentiation, whereas NP-specific deletion of *Smo* leads to healthy AF and CEP tissues. Taken together, our study convincingly demonstrated that Hh signaling is directly required by AF/CEP cells to maintain their cell phenotypes. Notably, abnormal differentiation of AF cells was also observed in mice lacking *Bmal1*, *Bgn*, *Foxo1/3/4*, or *Nfat5* [7, 9, 31, 37]. It will be interesting to determine whether Hh signaling maintains AF cell phenotypes by interacting with these proteins.

Prior studies have revealed a direct role for Hh signaling in the regulation of NP formation during embryonic development [14, 38]. Interestingly, Gli1 and Ptc1 proteins, two known indicators of Hh signaling activity, were also detected in the NP cells of early postnatal mice [14]. In addition, Gli1 protein was shown to be strongly expressed in the NP tissues of human and rat [19]. These results suggested that Hh signaling is activated in the postnatal NP tissues. In contrast to these studies, we here demonstrated that Gli1 is completely absent in the NP compartment of both juvenile and adult mice, indicating that Hh signaling is not active in the NP cells at these stages. The difference between our and other studies may be due to different approaches used to detect Gli1 expression. Additionally, this difference may reflect species- or stage-specific activity of Hh signaling in NP cells. Hh signaling may be active in NP tissues of human/rat and newborn mice, but inactive in NP tissues from 2-week-old or older mice. In addition to these expression analyses, the functional study of Hh signaling in NP cells by Liu et al. also yielded different results from ours. They found that *shRNA*-mediated knockdown of *Gli1* or pharmacological inhibition of Gli1/Gli2 by GANT61 in primary rat NP cells had deleterious effects on these cells, and therefore proposed that Hh signaling may play a cell-autonomous role in the regulation of postnatal NP cells similar to its function in NP development [19]. However, we showed that although *Agc1-CreER*^*T2*^*-*mediated deletion of *Smo* in disc cells of juvenile mice causes a defect in NP cell maturation in adult mice and accelerates age-related NP degeneration, NP-specific inactivation of Hh signaling doesnot result in any NP phenotype in either adult or aging mice. Therefore, our study provided strong genetic evidence to support Hh signaling regulating NP maturation and degeneration via a non-cell autonomous mechanism.

Postnatal NP undergoes a maturation process characterized by a change in morphology from a vacuolated sphere to a spindle-like shape with a corresponding increase in extracellular proteoglycan around the cells [35]. Mechanistically, this process has been shown to be regulated by the integrin αvβ6-TGFβ signaling cascade activated by physiological levels of mechanical stress [35]. Therefore, the impaired NP maturation in *Smo*^*Agcl*^ mice could be explained by the insufficient mechanical load caused by relatively moderate AF defects in these mice. On the other hand, the accelerated degeneration of NP during aging in *Smo* knockout mice may be related to prolonged excessive mechanical stress caused by severer AF defects. Indeed, a causal relationship between AF defects and NP degeneration has been previously observed in several loss-of-function studies [4, 9, 39]. In addition to AF defects, defects in other tissues, such as CEP, vertebral bone mass or vertebral growth plate, may also contribute to the NP phenotypes in *Smo*^*Agcl*^ mice. Another interesting finding in our study is that 4-month-old GDC-treated mice, but not age-matched *Smo*^*Agcl*^ mice, exhibited the degenerative changes in the NP compartment. The cause of this phenomenon remains to be determined. One possibility is that *Agc1-CreER*^*T2*^ is insufficient to effectively delete *Smo* in disc cells of juvenile mice. As a result, GDC treatment exerts more potent suppression of Hh signaling in the disc cells than *Smo* ablation. Additionally, it may imply that Hh signaling in cells that are not targeted by *Agc1-CreER*^*T2*^ but inhibited by GDC treatment plays an important role in maintaining homeostasis of postnatal NP tissues.

Appearance of chondrocyte-like cells is a common pathological feature of degenerated NP tissues in both mice and human [8]. Indeed, we also observed cells morphologically resembling hypertrophic chondrocytes in the NP tissue from 16-month-old *Smo*^*Agcl*^ mice. Similar cellular changes were also found in degenerated NP tissues of SM/J mice (an early-onset IDD mouse strain), naturally aged mice, and multiple knockout mice (such as *Bgn*, or *Foxo1/3/4, Tnmd*, *Nfat5*, or *Mkx* knockout mice) [4–6, 8, 9, 31]. However, the origin of chondrocyte-like cells in degenerated NP tissues remains controversial. It may result from the transformation of reticular NP cells, infiltration of adjacent AF/CEP cells, or both [8]. To definitely address this issue, elegant lineage tracing experiments using inducible Cre mouse lines specifically targeting NP or AF/CEP cells need to be performed. In addition, IDD has traditionally been associated with the reduction of proteoglycan in NP tissues. However, in our study and many others, significant increases in extracellular proteoglycan levels were observed in degenerated NP tissues [4–6, 8, 9, 31]. We believe that this difference may represent different degrees of IDD. Specifically, in the early stages of NP degeneration, enhanced chondrogenic events may occur in NP tissues, resulting in increased levels of proteoglycan and other cartilaginous proteins. These chondrogenic events may reflect the reparative effort of NP cells to combat the degenerative process of IVDs. In the later stages of NP degeneration, a large number of cells residing in the NP compartment may undergo apoptosis, resulting in fewer matrix-producing cells and ultimately a decrease in the proteoglycan matrix.

Hh signaling exerts it biological functions predominantly through Gli family of transcription factors, which include Gli1, Gli2, and Gli3 in mammals. The relative contribution of these Gli proteins to Hh function is known to be context-dependent [40, 41]. In this study, we showed that *Gli2* knockout mice did not show obvious degenerative changes in the IVDs, suggesting that it is not the major mediator of Hh signaling in discs cells. In contrast, a recent study showed that systemic administration of GANT61, an inhibitor of Gli1 and Gli2 [42], to rats resulted in smaller NP area and IVD height with minimal effects on the AF compartment [19]. Notably, these IVD phenotypes caused by GANT61 treatment are distinct from those exhibited in mice receiving GDC treatment or *Smo* ablation. Therefore, whether and how Gli1 and Gli2 play a redundant role in mediating Hh function in disc cells remain uncertain. On the other hand, our genetic rescue experiments demonstrated that Hh signaling regulates IVD growth and homeostasis primarily by decreasing Gli3R. However, we should acknowledge that in this study we only analyzed the IVD phenotypes of 4-month-old *Gli2* or *Gli3*-deficient mice, and therefore it remains to be determined whether the same Gli factor is responsible for mediating the function of Hh signaling in the pathogenesis of age-related IDD.

The mechanism regulating IVD growth and homeostasis downstream of Gli factors is yet to be elucidated. In this regard, Hh signaling has been shown to interact with Wnt/β-catenin in a variety of cell types, activating or suppressing Wnt/β-catenin signaling depending on the cellular context [29, 43]. Interestingly, β-catenin protein levels were found to be significantly up-regulated in the degenerated IVD tissues of both mice and humans [44–46], and sustained activation of Wnt/β-catenin signaling led to degenerative changes in IVDs in mice, including the enlargement of NP tissues accompanied by a significant reduction in proteoglycan levels, disorganization of AF structure, severe loss of CEP and vertebral GP, and ectopic formation of osteophytes [45–47]. Additionally, β-catenin activation induced LBP in mice. Thus, inhibiting Wnt/β-catenin may offer a potential therapeutic approach for the treatment of IDD and LBP. Indeed, CRISPR-Cas9-mediated deletion of β-catenin attenuated injury-induced IDD in mice [44], while inhibition of β-catenin/TCF interaction by iCRT14 reversed LBP in an IDD mouse model induced by lumbar spine instability (LSI) [46]. The similar disc phenotypes exhibited by mice with β-catenin activation and *Smo* deletion suggest that Hh signaling may also interact with Wnt/β-catenin signaling in the IVD tissues. We speculate that Hh signaling maintains IVD homeostasis by restraining excessive Wnt/β-catenin signaling in disc cells. Conversely, it is also possible that Hh signaling is regulated by Wnt/β-catenin signaling, and excessive Wnt/β-catenin promotes IDD by inhibiting Hh signaling. Clearly, further experiments are warranted to elucidate the precise relationship between Hh and Wnt/β-catenin signaling during IVD development and homeostasis as well as validate whether Hh signaling activation has the same therapeutic effects on IDD and LBP as β-catenin inhibition.

In summary, our study revealed that Hh signaling not only has a cell-autonomous function in the AF/CEP tissues, but also indirectly promotes NP cell maturation during IVD growth and prevents NP degeneration during aging. Mechanistically, Hh signaling controls IVD growth and homeostasis mainly by antagonizing the activity of Gli3 transcriptional repressor. Thus, Hh pathway may be targeted to prevent and treat IDD.

### Electronic supplementary material

Below is the link to the electronic supplementary material.

Supplementary Material 1

## Data Availability

The data that support the findings of this study are available upon reasonable request.
