# Supplementary material for: Intervertebral disc-intrinsic Hedgehog signaling maintains disc cell phenotypes and prevents disc degeneration through both cell autonomous and non-autonomous mechanisms
Source: Cell Mol Life Sci. 2024 Feb 3;81(1):74. doi: 10.1007/s00018-023-05106-x (PMC10838248; doi:10.1007/s00018-023-05106-x)
Supplement: Supplementary file 1 — Supplementary Material 1 [file 18_2023_5106_MOESM1_ESM.docx]

**
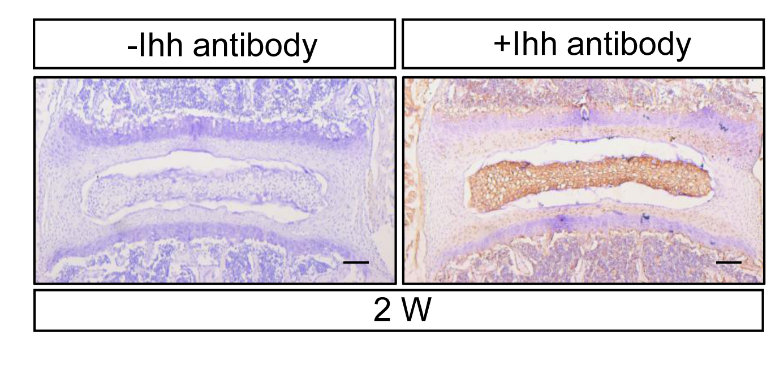
**

**Supplemental Figure 1.** Immunohistochemical (IHC) staining was used to evaluate the expression of Ihh in paraffin sections of lower lumbar IVDs in 2-week-old mice. The expression of Ihh is shown in brown. Scale bar: 200 μm.


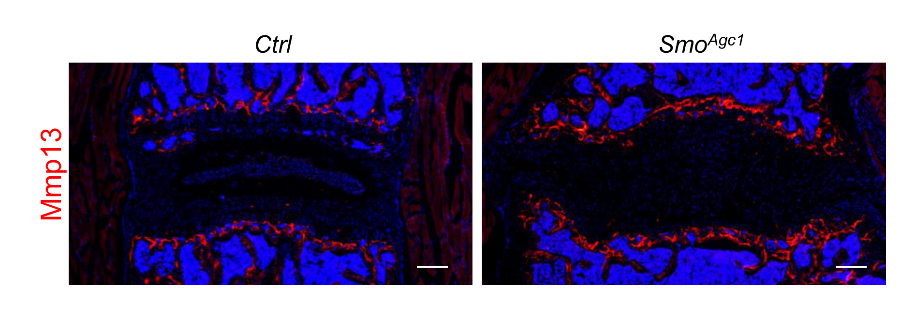


**Supplemental Figure 2.** Mmp13 immunofluorescent (IF) staining of frozen sections of lower lumbar IVDs from the above mice. The expression of Mmp13 is shown in red. All above *Ctrl* and *Smo^Agc1^* mice were injected with tamoxifen once daily for 5 days starting at 2 weeks of age. Scale bar: 200 μm.


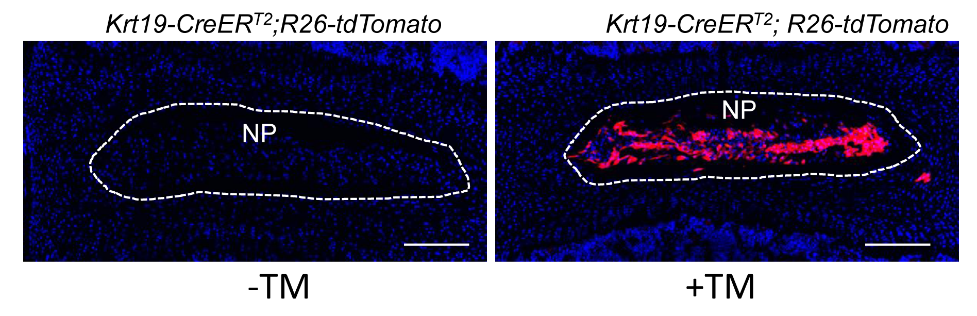


**Supplemental Figure 3.** Representative fluorescent images of frozen sections of lower lumbar IVD from *Krt19-CreER*; *R26-tdTomato* mice that were injected with corn oil (-TM) or tamoxifen (+TM) once daily for 5 days starting at 2 weeks of age and evaluated at 1 day after last injection. IVD cells targeted by *Krt19-CreER* appear red. NP: nucleus pulpous. The NP compartment is demarcated by white dashed lines. Scale bar: 200 μm.


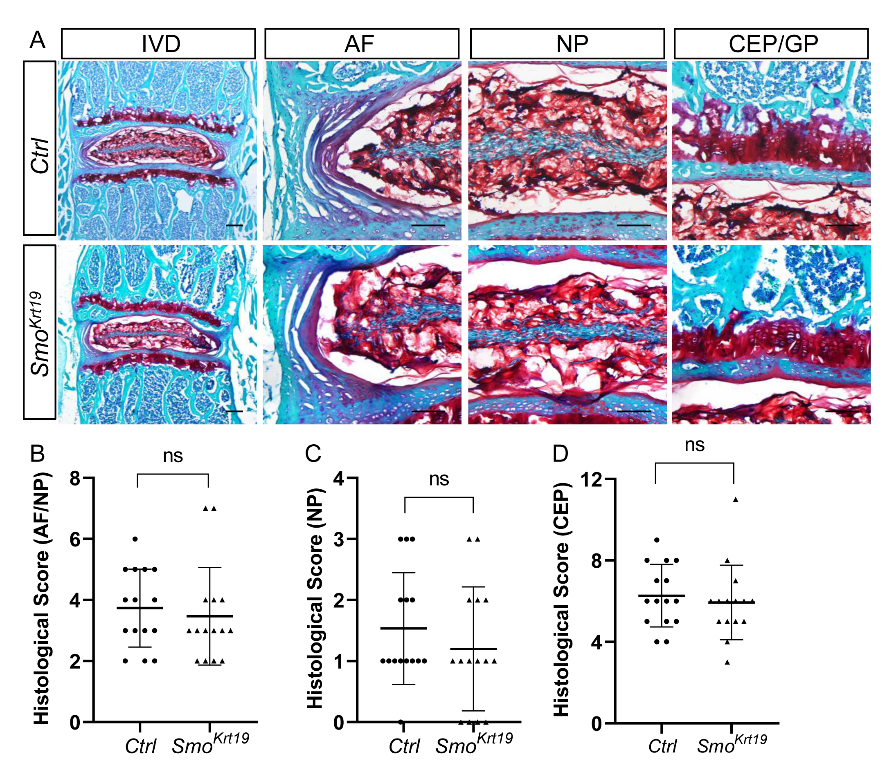


**Supplemental Figure 4.** (A) Safranin O/Fast green staining of the mid-coronal sections of lower lumbar IVDs from 16-month-old *Smo^c/c^ (Ctrl)* and *Krt19-CreER; Smo^c/c^* (*Smo^Krt19^*) mice. The left panel in each row is low-magnification images of the entire IVD and the other panels are high-magnified views. IVD: intervertebral disc; NP: nucleus pulposus; AF: annulus fibrosus; CEP: cartilaginous endplate; GP: vertebral growth plate. Scale bar: 200 μm in low-magnification images, 100 μm in high-magnification images. (B) Evaluation of histopathological scores for AF/NP, NP, and CEP in lower lumbar IVDs of the above mice. All above *Ctrl* and *Smo^Krt19^* mice were injected with tamoxifen once daily for 5 days starting at 2 weeks of age. n=15 IVDs (L4-L5, L5-L6, L6-S1) from 5 mice per genotype. Data were presented in dot-plots showing individual data points and their mean ± SD for each group. Each data point indicates a value from one IVD. Statistical significance was determined using Student’s t-test. *: *p* < 0.05; **: *p* < 0.01; ***: *p* < 0.001; ****: *p* < 0.0001.
